# Immune-Related Functions of the *Hivep* Gene Family in East African Cichlid Fishes

**DOI:** 10.1534/g3.113.008839

**Published:** 2013-10-18

**Authors:** Eveline T. Diepeveen, Olivia Roth, Walter Salzburger

**Affiliations:** * Zoological Institute, University of Basel, 4051 Basel, Switzerland; † Evolutionary Ecology of Marine Fishes, Helmholtz Centre of Ocean Research Kiel (GEOMAR), D-24105 Kiel, Germany

**Keywords:** positive selection, immune response, adaptive radiation, molecular evolution

## Abstract

Immune-related genes are often characterized by adaptive protein evolution. Selection on immune genes can be particularly strong when hosts encounter novel parasites, for instance, after the colonization of a new habitat or upon the exploitation of vacant ecological niches in an adaptive radiation. We examined a set of new candidate immune genes in East African cichlid fishes. More specifically, we studied the signatures of selection in five paralogs of the *human immunodeficiency virus type I enhancer-binding protein* (*Hivep*) gene family, tested their involvement in the immune defense, and related our results to explosive speciation and adaptive radiation events in cichlids. We found signatures of long-term positive selection in four *Hivep* paralogs and lineage-specific positive selection in *Hivep3b* in two radiating cichlid lineages. Exposure of the cichlid *Astatotilapia burtoni* to a vaccination with *Vibrio anguillarum* bacteria resulted in a positive correlation between immune response parameters and expression levels of three *Hivep* loci. This work provides the first evidence for a role of *Hivep* paralogs in teleost immune defense and links the signatures of positive selection to host–pathogen interactions within an adaptive radiation.

The interplay between hosts and their parasites (*i.e.*, macroparasites, bacteria, and viruses) represents one of the strongest biological interactions (Haldane 1949). Pathogens impose strong selection pressures on their hosts and have the potential to rapidly change the genotypic composition of host populations, which may ultimately alter the structure of entire ecosystems (Thompson 1988; Ebert and Hamilton 1996; Keesing *et al.* 2010). To counteract the permanently evolving pathogen virulence, hosts evolve resistance through diverse immune response mechanisms (Hamilton 1980). These include the discrimination between self and nonself, facilitating the recognition of pathogen-derived epitopes (Altizer *et al.* 2003; Boots *et al.* 2008). Invertebrates and vertebrates share the architecture of the innate immune system, *i.e.*, a conserved immediate defense mechanism including Toll-like receptors, lysozymes, and cellular defenses (Janeway *et al.* 2001). Immune memory, however, is the hallmark of the adaptive immune system of vertebrates (Cooper and Alder 2006; Flajnik and Kasahara 2010). The unique somatic diversification of receptors of the immunoglobulin family during ontogeny [*i.e.*, V(D)J recombination] mediates a dramatic increase in the number of foreign pathogen epitopes that the adaptive immune system can remember and attack (Janeway *et al.* 1996; Zhu *et al.* 2012).

Because of the constant interplay between host–parasite adaptation and counteradaption, immune genes are commonly characterized by signatures of positive selection through elevated rates of adaptive protein evolution (Hughes and Nei 1988; Hughes and Nei 1989; Jansa *et al.* 2003; Schlenke and Begun 2003; Sawyer *et al.* 2004; Nielsen *et al.* 2005; Jiggins and Kim 2007; Sackton *et al.* 2007; Tschirren *et al.* 2011). Selection on immune system diversification can be particularly strong when hosts encounter novel pathogens that induce primary immune challenges. This is the case after the colonization of a new habitat or upon the exploitation of vacant ecological niches (Scharsack *et al.* 2007; Matthews *et al.* 2010; Jones *et al.* 2012). For instance, it has been shown that migratory birds that encounter two or more different parasite faunas have larger immune defense organs (*e.g.*, bursa and spleen) than closely related resident birds (Møller and Erritzøe 1998). Freshwater sticklebacks differ in their immune competence potential depending on their ecotype (*e.g.*, lower parasite diversity in rivers than in lakes) (Scharsack *et al.* 2007), which is furthermore supported by a correlation between major histocompatibility complex (MHC) genotype and foraging habitat in benthic and limnetic ecomorphs (Matthews *et al.* 2010). Given the recognized evolutionary importance of the immune system (Janeway *et al.* 2001; Rodríguez *et al.* 2012) and the range of available functional and theoretical knowledge, the next step would be to assess to which degree immune genes contribute to, or even trigger, macroevolutionary events such as divergence, rapid speciation, and adaptive radiation.

East African cichlid fishes are a classic example of adaptive radiation (Schluter 2000). Because of their great numbers of closely related endemic species and their high levels of phenotypic and ecological diversity, cichlids are an important model system to study the genetic basis of diversification, adaption, and speciation (Kornfield and Smith 2000; Kocher 2004; Seehausen 2006; Salzburger 2009; Santos and Salzburger 2012). Previous studies of cichlid adaptive radiations have mainly focused on the understanding of ecologically important traits (and their genetic basis), such as the feeding apparatus (Terai *et al.* 2002b; Albertson *et al.* 2005; Fraser *et al.* 2008; Fraser *et al.* 2009; Muschick *et al.* 2011; Muschick *et al.* 2012), as well as on sexually selected traits such as coloration and pigmentation (Terai *et al.* 2002a; Terai *et al.* 2003; Salzburger *et al.* 2007; Roberts *et al.* 2009). Fewer studies have addressed the evolution of the immune system or, more generally, physiology in relation to diversification and rapid speciation in cichlids (Blais *et al.* 2007; Gerrard and Meyer 2007; Dijkstra *et al.* 2011). Dijkstra *et al.* (2011), for example, showed that divergence in coloration is accompanied by differentiation in immune function in Lake Victoria cichlids, and divergence in alleles of the MHC has previously been proposed as trigger of speciation in Lake Malawian cichlids through MHC-mediated mate choice (Blais *et al.* 2007). Several genes related to the immune system, including MHC loci, have been found to show signs of positive selection in East African cichlids (Gerrard and Meyer 2007), suggesting a role for immune genes during cichlid adaptive radiations.

In this study, we focused on the function and molecular characterization of a novel family of immune genes in (cichlid) fishes, which have been implicated to have immunological and developmental functions in mammals and insects (Seeler *et al.* 1994; Wu *et al.* 1996; Torres-Vazquez *et al.* 2001). In a previous study that focused on a candidate gene family for neural crest-derived structures in cichlids (*i.e.*, the endothelin family of ligands and receptors), we detected strong signatures of positive selection in a gene adjacent to one of the focal loci, the zinc finger protein *human immunodeficiency virus type I enhancer-binding protein 1* (*Hivep1*) (Diepeveen and Salzburger 2011). *Hivep1* is a transcription factor with functions in a variety of biological and developmental processes, *e.g.*, *HIV-1* gene expression (Maekawa *et al.* 1989; Muchardt *et al.* 1992; Seeler *et al.* 1994), in the *Decapentaplegic* signaling pathway important for cell fate specification during embryogenesis (Grieder *et al.* 1995; Dai *et al.* 2000; Marty *et al.* 2000; Torres-Vazquez *et al.* 2001), in V(D)J recombination of immunoglobulins (Wu *et al.* 1993; Wu *et al.* 1996), and in MHC enhancer binding (Baldwin *et al.* 1990; William *et al.* 1995). Although a single copy of this gene is found in *Drosophila*, mammals are typically characterized by three copies (Hicar *et al.* 2001; Dürr *et al.* 2004). Teleost fish, however, possess up to five duplicates (see Braasch *et al.* 2009), which is in accordance with the 3R hypothesis of a teleost-specific genome duplication event after the 2R duplications in the vertebrate linage (Sidow 1996; Taylor *et al.* 2003; Meyer and Van De Peer 2005; Volff 2005).

The goal of the current study was threefold. First, we characterized the signatures of selection (*i.e.*, *d*_N_/*d*_S_ ratios) in the five *Hivep* paralogs in 40 East African cichlid fish species to determine whether adaptive protein evolution is commonly observed in the *Hivep* gene family. To this end, we performed phylogenetic analyses of the *Hivep* loci and estimated *d*_N_/*d*_S_ ratios on both codon sites and in individual cichlid lineages. Second, we examined the role of the *Hivep* paralogs in the immune defense in the cichlid *Astatotilapia burtoni*. We evaluated the functional connection between *Hivep* expression levels and several cellular immune parameters after an experimental vaccination with *Vibrio anguillarum* bacteria. This fish pathogen was chosen to simulate a novel immune challenge, as the host was expected to be immunologically naïve against these *Vibrio* bacteria. Finally, we examined putative pleiotropic developmental functions through analyses of *cis*-regulatory regions to obtain insights into other functions of the *Hivep* paralogs in teleosts that could be linked to the observed signatures of adaptive protein evolution and related our findings to the explosive speciation events in East African cichlid fishes.

## Materials and Methods

### Sampling, DNA and RNA extraction, and housing conditions

Samples for the DNA analyses were collected during two expeditions to Lake Tanganyika in 2007 and 2008 using a standard operating procedure described by Muschick *et al.* (2012). In total, 40 different cichlid species from 14 different lineages, including all major cichlid lineages in East Africa (so-called tribes) (Muschick *et al.* 2012) were examined (Supporting Information, Table S1). RNA for the gene expression assays was extracted from gill, brain, and liver tissue of adult *A. burtoni* (laboratory strain, both sexes; see *Experimental Vaccination* section). DNA and RNA extractions were performed as described elsewhere (Diepeveen and Salzburger 2011), with one exception: the tissue homogenization during the RNA extraction was performed on a BeadBeater (FastPrep-24; MP). Animals being part of the experimental vaccination study were kept under standard conditions (12 hr light, 12 hr dark, 25°) in the animal facilities at the Zoological Institute in Basel before transportation to the Helmholtz Centre for Ocean Research Kiel, where they were kept under the following conditions: 14 hr light; 10 hr dark; and 25° for ≥38 hr before the start of the experimental vaccination.

### Loci, PCR amplification, and sequencing

Previously, five nuclear *Hivep* paralogs (*i.e.*, *Hivep1*, *Hivep2a*, *Hivep2b*, *Hivep3a*, and *Hivep3b*) were identified in teleost fishes (Braasch *et al.* 2009). Ensemble (versions 61 and 67) sequences from the following species were downloaded: zebrafish (*Danio rerio*); cod (*Gadus morhua*); medaka (*Oryzias latipes*); spotted green pufferfish (*Tetraodon nigroviridis*); fugu (*Takifugu rubripes*); tilapia (*Oreochromis niloticus*); and stickleback (*Gasterosteus aculeatus*) (Table S2). For two loci, we performed in-house tblastx searches on the server of the Zoological Institute (University of Basel) to determine Hivep protein sequences in the preliminary cichlid genomes of *A. burtoni*, *Neolamprologus brichardi*, and *Pundamilia nyererei* (BROAD Institute, unpublished data). These teleost and cichlid sequences were aligned with Codon Code Aligner 3.7.1 (CodonCode Corporation) to determine exon–intron structure and to design cichlid-specific primers (Table S3).

Subsequent PCR and sequencing reactions were performed as described elsewhere (Diepeveen and Salzburger 2011). PCR products were visualized with GelRed (Biotium) on a 1.5% agarose gel. Sequences were aligned and visually inspected using Codon Code Aligner 3.7.1 (CodonCode Corporation) and exon/intron boundaries were determined based on homology with the obtained other teleost sequences. Total sequenced regions (TSR), protein-coding regions, and concatenated (TSRs of all five loci) data sets were constructed. All generated cichlid *Hivep* sequences have been deposited into GenBank (GenBank KF049218**–**KF049416) (Table S1).

### Phylogenetic analyses and tests for selection pressure

Phylogenetic analyses and tests for selection pressure were performed as described elsewhere (Diepeveen and Salzburger 2011; Diepeveen *et al.* 2013). In short, models of nucleotide substitution were chosen based on likelihood ratio tests (LRTs) conducted in jModeltest 0.1.1 (Guindon and Gascuel 2003; Posada 2008) and used in maximum likelihood searches in PAUP* (Swofford 2002) and Bayesian Inference in MrBayes 3.2 (Huelsenbeck and Ronquist 2001; Ronquist and Huelsenbeck 2003) for each individual paralog and for the concatenated dataset. Bootstrap analyses with 100 replicates were performed in PAUP* and MrBayes was run for 10,000,000 generations. *Tylochromis polylepis* and/or *Oreochromis tanganicae* were used as the outgroup in these analyses (Salzburger *et al.* 2002). The consensus tree based on the concatenated dataset was used as a common input tree in the subsequent analyses.

Both site and branch-site models, as implemented in Codeml, Phylogenetic Analysis by Maximum Likelihood (PAML) 4.2 (Yang 1997; Yang 2007), were used to test for selection pressure. The nonsynonymous/synonymous substitution rate ratio, *ω* or *d*_N_/*d*_S_, the proportion of sites assigned to an *ω* category, the *p_0,1,2_*, and the *p* and *q* parameters of the *β* distribution were estimated for all five datasets under different models. LRTs of the following model comparisons were performed to detect sites under positive selection: M1a (nearly neutral) with M2a (positive selection); M7 (β) with M8 (β and ω_s_ ≥1); and M8a (β and ω_s_ = 1) with M8. The comparison between M0 (one ratio) and M3 (discrete) was used as a test of variable *ω* among sites. Posterior probabilities for site classes were calculated with the Bayes empirical Bayes (BEB) (Yang *et al.* 2005). Next, LRTs between the null model (ω_s_ = 1) and the alternative model (ω_s_ ≥1) were performed to determine if focal, or foreground, lineages evolved under non-neutral evolution. These foreground branches were chosen based on the results from the phylogenetic and PAML analyses.

Subsequent sliding window analyses (window size = 20) were performed with TreeSAAP (selection on amino acid properties) 3.2 (Woolley *et al.* 2003) for the four loci for which positively selected sites were observed with the PAML analyses (*Hivep1*, *Hivep2b*, *Hivep3a*, and *Hivep3b*). Amino acids were categorized based on 31 physicochemical properties to identify regions of positive selection. Selection on amino acids was subsequently screened for positive destabilizing selection by means of categorizing the substitutions into eight categories (categories 1–8) based on the magnitude of radicality (*i.e.*, 1 is the most conservative amino acid substitutions and 8 is the most radical). The three highest categories (6–8; *P* ≤ 0.001) were used as indicative of radical amino acid substitutions. Next, these substitutions were analyzed with the program SIFT (sorting intolerant from tolerant) (Ng and Henikoff 2003) to screen for possible effect on protein function.

### Analyzing *cis*-regulatory regions

The five *Hivep* sequences from *A. burtoni* were compared with the obtained teleost sequences of *O. niloticus*, *O. latipes*, *T rubripes*, *T. nigroviridis*, and *D. rerio* with mVISTA (Mayor *et al.* 2000; Frazer *et al.* 2004). Sequences were globally aligned with Shuffle-LAGAN (Brudno *et al.* 2003) and the minimum sequence similarity was set to 50%. Intragenic conserved noncoding elements were predicted and analyzed with rVISTA (Loots *et al.* 2002) to identify potential transcription factor binding sites.

### Experimental vaccination and immune response measurements

To examine the expression patterns of the *Hivep* paralogs after an experimental vaccination, we exposed adult cichlid fish of the species *A. burtoni* to *Vibrio* bacteria following Roth *et al.* (2011). *V. anguillarum* was physically isolated from the stomach of freshly caught broad-nosed pipefish (*Syngnathus typhle*) (Roth *et al.* 2012). Strain confirmation was performed via sequencing of the *16S rRNA*, *recA*, and *pyrH* loci (GenBank reference numbers provided in Roth *et al.* 2012). On day 1 of the experiment, fish of both sexes were randomly assigned to either the experimental treatment (12 individuals) or the control treatment (11 individuals) and injected intraperitoneally with either 50 µl heat-killed (60 min at 65°) *V. anguillarum* (phylotype S6M4; 10^6^ cells/ml dissolved in phosphate-buffered saline (PBS), *i.e.*, experimental treatment) or 50 µl PBS (*i.e.*, control treatment), respectively, according to the methods of Roth *et al.* (2012). Fish were tagged subcutaneously with visible implant elastomer tags (Northwest Marine Technology) according to treatment and kept in a single aquarium system. After ∼21 hr of exposure, fish were killed with MS222 and weight and standard length were noted as in Birrer *et al.* (2012). Blood was collected from the caudal vessel in heparinized capillaries (Na-heparinized; Brand GMBH + Co. KG), followed by extraction of the head kidneys and spleen, which were forced through a 40-µm nylon sieve to prepare cell suspensions for subsequent cellular immune measurements. All steps were performed on ice. Cells were washed twice with RPMI medium (10 min, 600 rpm, 4°) and resuspended in a final volume of 450 µl.

The number of lymphocytes and monocytes (as proxies for immune response in the form of inflammation and/or stress to the treatment) were measured in blood, head kidneys, and spleen tissues by means of flow cytometry (FACSCalibur; Becton Dickinson) with pre-assessed cichlid-specific settings for each tissue type. The proportions of monocytes, lymphocytes, and the lymphocyte/monocyte ratio were calculated. Furthermore, the activity of the relative number of lymphocytes in the G_2-M_ and synthesis (S) phases of the proliferation cycle compared to the relative number of lymphocytes in the G_0-1_ phase was measured by killing cells in ethanol and subsequent labeling of the DNA with propidium iodide (Sigma Aldrich) as described by Roth *et al.* (2011). Lymphocytes were identified by their characteristic FSC/SSC pattern (*i.e.*, cell volume and inner complexity). Proliferating cells in the G_2-M_ phase were distinguished from G_0-1_ and S phase cells by a more intense red fluorescence because of their higher DNA content. To test whether the obtained data were normally distributed, D’Agnostino and Pearson omnibus normality tests as implemented in graphPad Prism version 5.0a for Mac OS X (http://www.graphpad.com) were conducted. Outliers with values outside 2 SDs from the mean were removed (*i.e.*, up to three individuals per treatment group and tissue type).

The experiment was performed according to current national legislation of the Ministerium für Landwirtschaft, Umwelt und ländliche Räume des Landes Schleswig-Holstein (project entitled “Effects of global change on the immunological interaction of pipefish and cichlids with their natural bacteria communities”). One fish from the control treatment died during the experiment.

### Gene expression assays and analyses

Gill, brain, and liver tissues of the 22 experimental animals were extracted and directly stored in RNA later (Invitrogen). RNA extraction and reverse-transcriptase were conducted as described elsewhere (Diepeveen and Salzburger 2011). Subsequent gene expression analyses were performed by means of quantitative PCR on a BioMark HD system at the Genetic Diversity Centre of the ETH Zurich, following the manufacturer’s protocol. Levels of gene expression were measured in 48.48 dynamic array integrated fluidic circuits with EvaGreen DNA binding dye. Primers were designed and tested for the five focal *Hivep* loci, two housekeeping loci [*i.e.*, elongation factor 1 (*EF1*) and ribosomal protein SA 3 (*RpSA3*)] (Colombo *et al.* 2013) and four control loci with demonstrated immune-related functions [*i.e.*, allograft inflammatory factor 1 (*AIF1*), anti-inflammatory response (Watano *et al.* 2001); coagulation factor II receptor-like 1 (*F2RL1*), inflammation and immunity (Rothmeier and Ruf 2012); interleukin 10 (*IL10*), immunosuppression (Jankovic and Trinchieri 2007); Toll-like receptor 5 (*TLR5*), pathogen recognition (Janeway and Medzhitov 2002; Akira *et al.* 2006)] (Table S3). Data were visualized, amplification plots were checked, and outliers were removed with the Fluidigm Real-Time PCR analysis software version 3.1 (Fluidigm). Further comparative analyses were performed with the qBase^PLUS2^ software package (Biogazelle). *EF1* and *RpSA3* were used as reference targets for the multiple reference gene normalization approach as implemented in qBase^PLUS2^ software (Biogazelle). Three different positive controls were included in this study; RNA was extracted from whole nonexperimental *A*. *burtoni* juveniles and two mixes composed of nine samples (gill, liver, and brain tissues of three randomly chosen individuals) each for the control group and the experimental group separately. Variation between PCR replicates and deviation of normalization factors were checked and outliers with values outside 2 SDs from the mean were removed. Data were controlled for inter-run variation.

Unpaired *t* tests were performed for the control immune genes expression levels between the control and experimental treatment groups with the qBase^PLUS2^ software (Biogazelle). To test for a correlation between the expression of *Hivep* paralogs [*i.e.*, quality-controlled and normalized relative quantities (CNRQ, here just RQ)] and the immune response measurements, Pearson correlations were calculated in GraphPad Prism in the control and experimental treatment groups.

## Results

### Phylogenetic analyses of cichlid *Hivep* sequences

**Figure 1:**
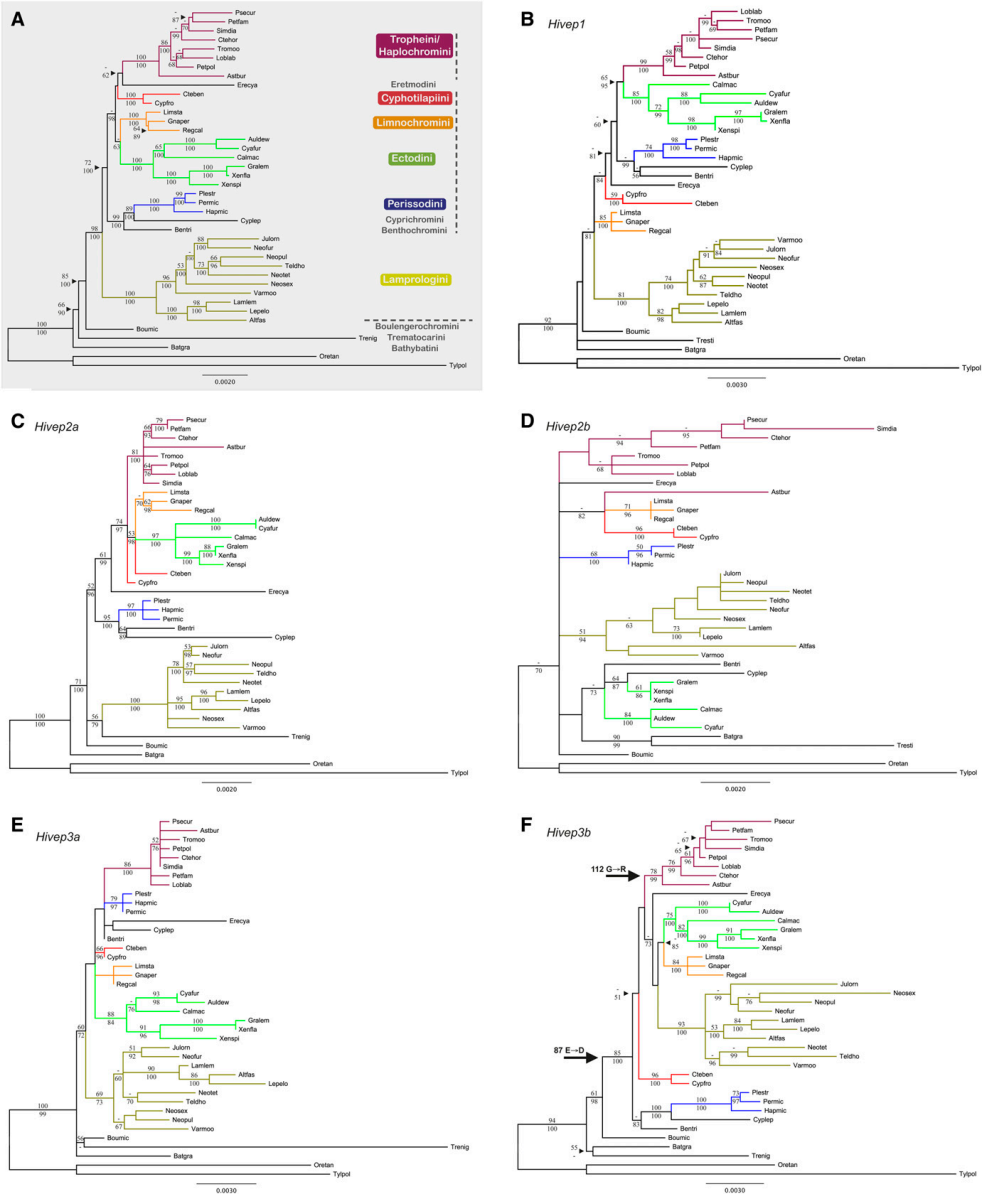
Phylogenetic hypotheses based on maximum likelihood for the concatenated dataset and the individual *Hivep* loci consisting of 40 taxa. (A) Concatenated dataset (13,543 base pairs (bp); best-fitting model of nucleotide substitution: HKY+I+G). Lineages are recovered with maximum support values, whereas relationships within and between lineages are supported with relative high values. The horizontal dotted line separates the five most basal species from the derived lineages: the lamprologines, the eretmodines, and the species belonging to the C-lineage, with the latter marked by the vertical dotted line. (B) *Hivep1* (3440 bp; TPM1uf+I+G) well-resolved with all major lineages recovered with high support values. (C) *Hivep2a* (3143 bp; TIM2+G). The lamprologines plus the five most basal species are found basal of the C-lineage plus Eretmodini. All major lineages are monophyletic, except the Cyphotilapiini. (D) *Hivep2b* (1517 bp; TrN+I+G). Mostly unresolved tree with a basal polytomy, excluding the two outgroup species from all other species. Polytomous relationships were further found for the haplochromine and ectodine lineages. (E) *Hivep3a* (2142 bp; TPM1uf+G). The lamprologines plus the five most basal species are found basal of the C-lineage plus Eretmodini. (F) *Hivep3b* (3301 bp; HKY+I+G). The lamprologines are positioned within the C-lineage. Black arrows represent the two branches for which *ω* > 1 was found in the branch-site analyses and their lineage-specific amino acid substitutions. Bootstrap values (PAUP*) and Bayesian posterior probabilities (MrBayes) >50% are shown, respectively, above and below the branches. Cichlid lineage names and a color key for the six cichlid lineages with more than one species included in this study are provided in the gray box in (A). Abbreviations of species names consist of the first three characters of the genus name followed by the first three characters of the species name (Table S1 shows full species names). Branch lengths of *T. polylepis* were shortened by 50% in all phylogenies and for *T. nigrifrons* in (E).

To examine the molecular evolutionary history of the *Hivep* paralogs, we performed maximum likelihood and Bayesian Inference phylogenetic analyses based on the total sequenced region per locus, and the concatenated dataset including all loci. The phylogenetic topologies of the obtained partial cichlid *Hivep* gene sequences and the concatenated dataset of 13.5 kb are displayed in Figure 1, A–F. Generally, the observed topologies of the gene trees, and the concatenated tree in particular, correspond with the available species trees (Salzburger *et al.* 2002; Takahashi 2003; Salzburger and Meyer 2004; Clabaut *et al.* 2005; Salzburger *et al.* 2005; Muschick *et al.* 2012), with *T. polylepis*, *O. tanganicae*, *B. graueri*, *B. microlepis*, and *T. nigrifrons* as most basal species, followed by the Lamprologini, the Eretmodini, and the species belonging to the "C-lineage" (Salzburger *et al.* 2002; Clabaut *et al.* 2005; Day *et al.* 2008) (Figure 1). As previously observed (Diepeveen and Salzburger 2011), *E. cyanostictus* was found at a different position within the C-lineage, whereas this species has been commonly resolved outside the C-lineage in previous studies (Salzburger *et al.* 2002; Clabaut *et al.* 2005; Day *et al.* 2008). Also, the relationships between the individual lineages of the C-lineage altered between the individual gene trees. Long branches were observed for *T. polylepis*, the lamprologines, individual lamprologine species, different branches within the ectodines in several gene trees, and for the haplochromines in *Hivep2b* and *T. nigrifrons* in the *Hivep3a* gene tree.

### Selection pressure on sites and branches

To investigate signatures of selection pressure in the *Hivep* paralogs, we used site and branch-site models (Yang 1997, 2007) to obtain, *e.g.*, nonsynonymous/synonymous substitution rate ratios (*d*_N_/*d*_S_). The maximum likelihood parameter estimations for *ω*, *p_0,1,2_* and *p*, and *q* under different evolutionary models can be found in Table 1 for all five *Hivep* loci. Estimations of *ω* under the one ratio model (M0) suggest that the *Hivep* genes evolved under purifying selection, with *ω* ranging from 0.093 (*Hivep2a*) to 0.303 (*Hivep1*). A small proportion of sites, 1.4% (*Hivep2a*) to 11.8% (*Hivep3b*), was estimated to have evolved neutrally (*ω* = 1) under the neutral model (M1a). By using models that allow *ω* to vary among sites (M2a, M3, and M8), up to 4.3% of sites were detected to have evolved with *ω* > 1 in *Hivep1*, *Hivep2b*, *Hivep3a*, and *Hivep3b*, with more than 89.4% of sites evolving under purifying selection.

**Table 1:** Site model parameter estimates for the five *Hivep* paralogs

	Parameter Estimates Using Different Models						
Locus	M0 (One Ratio)	M1a (Neutral)*^a^*	M2a (Selection)*^a^*	M3 (Discrete)*^a^*	M7 (β)*^a^*	M8 (β and ω)*^a^*	M8a (β and ω_s_ = 1)*^a^*
*Hivep1*	*ω* = 0.303	*p_0_* = 0.896, *ω_0_* = 0.000	*p_0_* = 0.899, *ω_0_* = 0.012	*p_0_* = 0.997, *ω_0_* = 0.106	*p* = 0.005, *q* = 0.046	*p* = 0.013, *q* = 0.091	*p* = 0.005, *q* = 4.470
		*p_1_* = 0.104, *ω_1_* = 1.000	*p_1_* = 0.099, *ω_1_* = 1.000	***p_1_* = 0.003, *ω_1_* = 18.416**		*p_0_* = 0.998	*p_0_* = 0.896
			***p_2_* = 0.002, *ω_2_* = 21.464**	***p_2_* = 0.000, *ω_2_* = 62.335**		***p_1_* = 0.002, *ω* = 21.496**	*p_1_* = 0.104, *ω* = 1.000
*Hivep2a*	*ω* = 0.093	*p_0_* = 0.986, *ω_0_* = 0.080	*p_0_* = 0.986, *ω_0_* = 0.080	*p_0_* = 0.543, *ω_0_* = 0.000	*p* = 0.554, *q* = 5.217	*p* = 0.554, *q* = 5.221	*p* = 0.672, *q* = 6.412
		*p_1_* = 0.014, *ω_1_* = 1.000	*p_1_* = 0.001, *ω_1_* = 1.000	*p_1_* = 0.270, *ω_1_* = 0.203		*p_0_* = 1.000	*p_0_* = 0.999
			*p_2_* = 0.012, *ω_2_* = 1.000	*p_2_* = 0.186, *ω_2_* = 0.203		*p_1_* = 0.000, *ω* = 1.00	*p_1_* = 0.001, *ω* = 1.000
*Hivep2b*	*ω* = 0.120	*p_0_* = 0.942, *ω_0_* = 0.000	*p_0_* = 0.941, *ω_0_* = 0.000	*p_0_* = 0.957, *ω_0_* = 0.000	*p* = 0.005, *q* = 0.049	*p* = 0.010, *q* = 0.160	*p* = 0.005, *q* = 2.777
		*p_1_* = 0.058, *ω_1_* = 1.000	*p_1_* = 0.059, *ω_1_* = 1.000	***p_1_* = 0.043, *ω_1_* = 1.439**		*p_0_* = 1.000	*p_0_* = 0.942
			***p_2_* = 0.000, *ω_2_* = 38.425**	***p_2_* = 0.000, *ω_2_* = 38.861**		***p_1_* = 0.000, *ω* = 38.446**	*p_1_* = 0.058, *ω* = 1.000
*Hivep3a*	*ω* = 0.251	*p_0_* = 0.919, *ω_0_* = 0.000	*p_0_* = 0.909, *ω_0_* = 0.000	*p_0_* = 0.984, *ω_0_* = 0.040	*p* = 0.005, *q* = 0.046	*p* = 0.005, *q* = 0.049	*p* = 0.005, *q* = 0.047
		*p_1_* = 0.081, *ω_1_* = 1.000	*p_1_* = 0.090, *ω_1_* = 1.000	***p_1_* = 0.016, *ω_1_* = 4.264**		*p_0_* = 0.999	*p_0_* = 1.000
			***p_2_* = 0.001, *ω_2_* = 31.219**	***p_2_* = 0.000, *ω_2_* = 47.201**		***p_1_* = 0.001, *ω* = 31.868**	*p_1_* = 0.000, *ω* = 1.000
*Hivep3b*	*ω* = 0.277	*p_0_* = 0.882, *ω_0_* = 0.000	*p_0_* = 0.894, *ω_0_* = 0.014	*p_0_* = 0.000, *ω_0_* = 0.000	*p* = 0.005, *q* = 0.046	*p* = 0.023, *q* = 0.170	*p* = 0.005, *q* = 2.855
		*p_1_* = 0.118, *ω_1_* = 1.000	*p_1_* = 0.102, *ω_1_* = 1.000	*p_1_* = 0.992, *ω_1_* = 0.090		*p_0_* = 0.996	*p_0_* = 0.882
			***p_2_* = 0.004, *ω_2_* = 13.855**	***p_2_* = 0.008, *ω_2_* = 10.193**		***p_1_* = 0.004, *ω* = 13.814**	*p_1_* = 0.118, *ω* = 1.000

The maximum likelihood parameter estimations for *ω*, *p_0,1,2_* and *p* and *q* under different evolutionary models for all five *Hivep* loci. Estimations of *ω* under the one ratio model (M0) suggest that the *Hivep* genes evolved under purifying selection. A small proportion of sites was estimated to have evolved neutrally (*ω* = 1) under the M1a model in all loci. By using the M2a, M3, and M8 models, a small proportion of sites were detected to have evolved under *ω* > 1 in *Hivep1*, *Hivep2b*, *Hivep3a*, and *Hivep3b*. *Hivep*, *human immunodeficiency virus type I enhancer-binding protein*.

a p_0-2_ are the proportions of sites assigned to the *ω* category or to a beta distribution with p and q as parameters; *ω* ratios >1 and their corresponding proportions are depicted in bold.

Likelihood ratio tests of several model comparisons (Table 2) were performed to detect positively selected amino acids. This approach resulted in the rejection of the null models in the M1a *vs.* M2a, M7 *vs.* M8, and M8a *vs.* M8 comparisons for all loci except *Hivep2a*. Positively selected sites were detected with the BEB for *Hivep1* (16 sites), *Hivep2b* (2 sites), *Hivep3a* (18 sites), and *Hivep3b* (13 sites).

**Table 2:** LRT statistics of three site model comparisons and positively selected sites

Locus	Test	LRT (2∆*l*)	*P*	Selected Sites (BEB)*
*Hivep1*	M1a *vs.* M2a	210.047	**<0.001**	**37S**, **49Q**, **61V**, **81N**, *106T*, *114R*, *130T*, **248A**, **292Q**, **472Q**, *530V*, *546L*, **558T**, **582P**, *587H*, **656N**
	M7 *vs.* M8	210.084	**<0.001**	**37S**, **49Q**, **61V**, **81N**, *106T*, *114R*, **130T**, **248A**, **292Q**, **472Q**, **530V**, **546L**, **558T**, **582P**, *587H*, **656N**
	M8a *vs.* M8	210.024	**<0.001**	See M7 *vs.* M8 comparison
*Hivep2a*	M1a *vs.* M2a	0.000	1.000	0
	M7 *vs.* M8	0.000	1.000	0
	M8a *vs.* M8	0.007	0.932	0
*Hivep2b*	M1a *vs.* M2a	35.537	**<0.001**	**143P**
	M7 *vs.* M8	37.555	**<0.001**	**143P**, *330Q*
	M8a *vs.* M8	34.250	**<0.001**	See M7 *vs.* M8 comparison
*Hivep3a*	M1a *vs.* M2a	304.843	**<0.001**	**62S**, **63A**, **64A**, *82S*, **141A**, **200S**, **265I**, *329Q*, **343P**, *371D*, **436V**, **445A**, **484T**, **565E**, *573T*, **703P**
	M7 *vs.* M8	307.001	**<0.001**	**62S**, **63A**, **64A**, *82S*, **141A**, *168N*, **200S**, **265I**, *329Q*, **343P**, *371D*, **436V**, *437K*, **445A**, **484T**, **565E**, *573T*, **703P**
	M8a *vs.* M8	307.003	**<0.001**	See M7 *vs.* M8 comparison
*Hivep3b*	M1a *vs.* M2a	111.745	**<0.001**	**87E**, **112G**, *218H*, **286T**, **326S**, *335I*, **352G**, *392G*, **399P**, *401P*, **403R**, **447I**, **511K**
	M7 *vs.* M8	113.003	**<0.001**	**87E**, **112G**, *218H*, **286T**, **326S**, **335I**, **352G**, *392G*, **399P**, **401P**, **403R**, **447I**, **511K**
	M8a *vs.* M8	111.749	**<0.001**	See M7 *vs.* M8 comparison

LRTs resulted in the rejection of the null models in the M1a *vs.* M2a, M7 *vs.* M8, and M8a *vs.* M8 comparisons for all loci except *Hivep2a*. Positively selected sites were detected for *Hivep1*, *Hivep2b*, *Hivep3a*, and *Hivep3b*. LRT, likelihood ratio test; BEB, Bayes empirical Bayes; *Hivep*, *human immunodeficiency virus type I enhancer-binding protein*.

a *P* = 0.01 (bold) and *P* = 0.05 (italic).

LRTs of the branch-site analyses were performed to test whether focal lineages evolved under non-neutral evolution. Significant LRTs were only observed for *Hivep3b* (Table 3), indicating that although the *ω* ratios do vary among sites for three of the other four *Hivep* loci, they do not seem to vary significantly among lineages. For *Hivep3b*, the following two branches were observed with *ω* > 1: the derived lineages (excluding the five basal species; *P* < 0.001) and the haplochromines (*P* = 0.031) (Table 3 and Figure 1).

**Table 3:** Parameter estimations and LRTs for the null and alternative hypotheses of the branch-site model for two different cichlid lineages for *Hivep3b*

Clade	Model	Site Class	0	1	2a	2b	LRT (*P*)
DL	Model A (Null)	Proportion	0.766	0.123	0.096	0.015	
		Background ω	0.000	1.000	0.000	1.000	
		Foreground ω	0.000	1.000	1.000	1.000	
	Model A (Alternative)	Proportion	0.897	0.087	0.014	0.001	18.509 (<0.001)
		Background ω	0.070	1.000	0.070	1.000	
		Foreground ω	0.070	1.000	7.974	7.974	
HC	Model A (Null)	Proportion	0.725	0.167	0.088	0.020	
		Background ω	0.000	1.000	0.000	1.000	
		Foreground ω	0.000	1.000	1.000	1.000	
	Model A (Alternative)	Proportion	0.806	0.185	0.008	0.002	4.628 (0.031)
		Background ω	0.000	1.000	0.000	1.000	
		Foreground ω	0.000	1.000	24.33	24.33	

LRTs of the branch-site analyses indicate that *Hivep3b* evolved under non-neutral evolution (*ω* > 1) in the following two focal lineages: the derived lineages (excluding the five basal species) and the haplochromines. LRT, likelihood ratio test; *Hivep*, *human immunodeficiency virus type I enhancer-binding protein*; DL, derived lineage; HC, Haplochromini.

### Sliding windows and amino acid substitution characteristics

**Figure 2:**
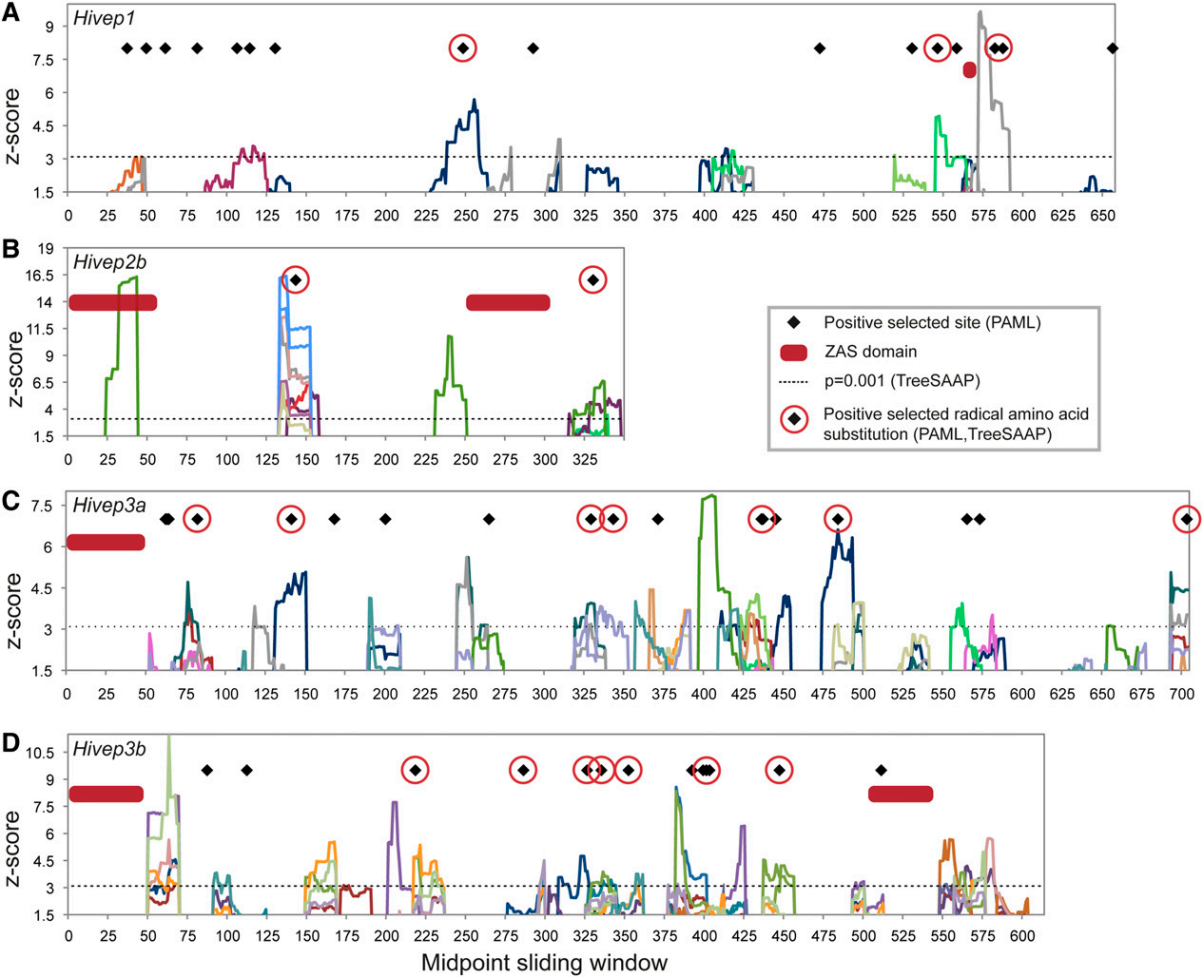
Sliding window plots and radical amino acid properties for four *Hivep* paralogs. (A) For *Hivep1*, multiple sliding windows were observed with z-score ≥ 3.09. For several positively selected sites identified with PAML, no radical substitutions were found and *vice versa*. (B) In *Hivep2b*, four regions of positive selection are observed of which two correspond with positively selected sites identified by the PAML analyses. (C) The observed regions of positive selection for *Hivep3a* are characterized by many different physicochemical properties. (D) Many radical amino acid substitutions were found for *Hivep3b* that correspond with most of the positively selected sites identified by PAML analyses. Each physicochemical amino acid property is individually color coded (see Figure S2 for details). Black diamonds (♦) represent positively selected amino acid sites obtained by the PAML analyses and red circles around them represent positively selected radical nonsynonymous substitutions (category 6-8). The dotted line at z-score = 3.09 represents *P* = 0.001, whereas z-score = 1.64 represents *P* = 0.05. Red rectangles represent the following ZAS domains: Hivep1 ZAS-C; Hivep2b ZAS-N, ZAS-C; Hivep3a ZAS-N; and Hivep3b ZAS-N, ZAS-C.

To visualize regions with elevated *d*_N_/*d*_S_ values and to connect such regions with the physiochemical properties of the respective amino acid substitutions, we performed sliding window analyses. The sliding window plots of *Hivep1*, *Hivep2b*, *Hivep3a*, and *Hivep3b* are depicted in Figure 2, A–D. Regions of positive selection (z-score ≥ 3.09 corresponding with *P* ≤ 0.001) were observed for all four loci, with highest z-scores for *Hivep2b*, and the most numerous regions with a z-score ≥ 3.09 observed for *Hivep3a*. Interestingly, not all of these retrieved regions of positive selection correspond with the obtained positively selected sites as identified by the PAML analyses and *vice versa*. Relative few regions of positive selection are observed in the ZAS domains that contain the zinc fingers that bind specific DNA motifs. Notable exceptions are the ZAS-N domain of *Hivep2b* and the ZAS-C domain of *Hivep3b* (Figure 2); the latter is furthermore characterized by a positively selected site identified by the PAML analyses. Most commonly observed positively selected amino acid properties among the four paralogs affect the alpha-helical tendencies, the compressibility, the equilibrium constant (ionization of COOH), and the surrounding hydrophobicity. The SIFT analyses of the observed substitutions to screen for possible effect on protein function showed that all substitutions are tolerant and thus have no predicted damaging effect on function (data not shown).

### Analyzing *cis*-regulatory regions

We investigated noncoding regions within the *Hivep* paralogs for potential *cis*-regulatory elements to determine possible binding sites for transcription factors, indicative of putative functional involvement in signaling pathways. Vistaplots of *Hivep1*, *Hivep2a*, *Hivep2b*, and *Hivep3b* are depicted in Figure S1, A–D. Because of a limited number of retrieved teleost sequences for *Hivep3a*, the Vistaplot was not informative for this locus and was therefore excluded from further analyses. For all four analyzed loci, conserved noncoding elements (CNEs) were observed in *A*. *burtoni*. Interestingly, a similar pattern of two CNEs surrounding a single exon was observed in all loci (arrows in Figure S1). Although this pattern seems common among teleost fish for *Hivep1* and *Hivep2a*, for *Hivep2b* and *Hivep3b* this pattern seems to be restricted to cichlid fishes (*O*. *niloticus* is the reference sequence in these analyses). A third cichlid-specific CNE was observed in a subsequent intron in both *Hivep1* and *Hivep2a*, whereas for *Hivep3b* two more cichlid-specific CNEs were identified.

Because the particular pattern of two CNEs surrounding an exon was observed in all four analyzed loci, the subsequent search for potential transcription factor binding sites was mainly focused on these regions to determine any overlap in possible function of these regions. The analyses resulted in similar hits among *Hivep* paralogs and suggested a possible association between the *Hivep* paralogs and different types of signaling pathways involved in, *e.g.*, sex determination [*androgen receptor* (*AR*); pre-B-cell leukemia transcription factor 1 (*PBX1*); sex-determining region Y protein (*SRY*)], immune system [*B-cell lymphoma 6 protein* (*BCL6*); H2.0-like homeobox protein (*HB24*); signal transducer and activator of transcription1,3,5a (*STAT1*,*3*,*5a*)], developmental patterning [homeobox protein Hox-A3 (*Hoxa3*); homeobox protein MSX-1 (*Msx1*)], and several members of the paired box protein Pax (*PAX*) and bone morphogenetic protein (*BMP*) pathways.

### Experimental vaccination and immune response measurements

**Figure 3:**
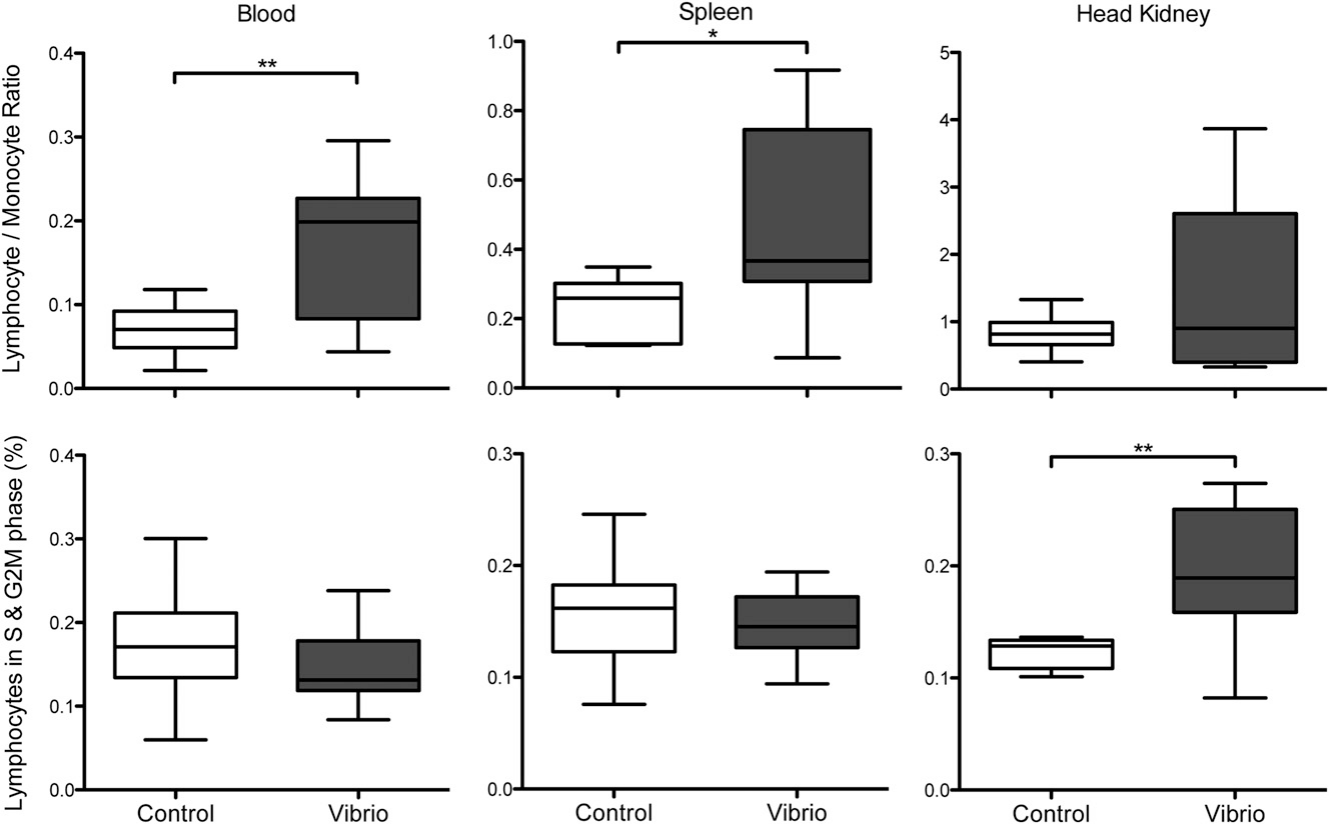
Immune response measurements after the experimental vaccination in *A. burtoni* adults. Lymphocyte/monocyte ratios (top) and proportions of cells in the S and G_2-M_ phases (bottom) measured in blood (left), spleen (center), and head kidney (right) for the control treatment (white boxplot) and experimental treatment (*Vibrio*; gray boxplot). *P < 0.05; **P < 0.01. Depicted are the median, lower and upper quartiles (box), and the minimum and maximum observed values (error bars).

We performed experimental vaccinations to test whether the *Hivep* paralogs are involved in an induced immune response. The experimental vaccination was realized by exposure to heat-killed *V. anguillarum* for ∼21 hr, following the methods of Birrer *et al.* (2012). Several immune response measurements were performed to determine induction of immune defense dynamics. The lymphocyte/monocyte ratio and the relative number of lymphocytes in the G_2-M_ and S phases of the proliferation cycle were measured in blood, spleen, and head kidney (Figure 3). Data were normally distributed. The experimental treatment resulted in an elevated lymphocyte/monocyte ratio in blood (*P* = 0.008; unpaired *t* test) and spleen (*P* = 0.018), indicative of a higher proportion of cells from the adaptive immune system (*i.e.*, immune response). A higher proportion of lymphocytes in the S and G_2-M_ phases was found in the head kidney of the experimental group (*P* = 0.005), indicative of induced lymphocyte proliferation.

### Gene expression assays

**Figure 4:**
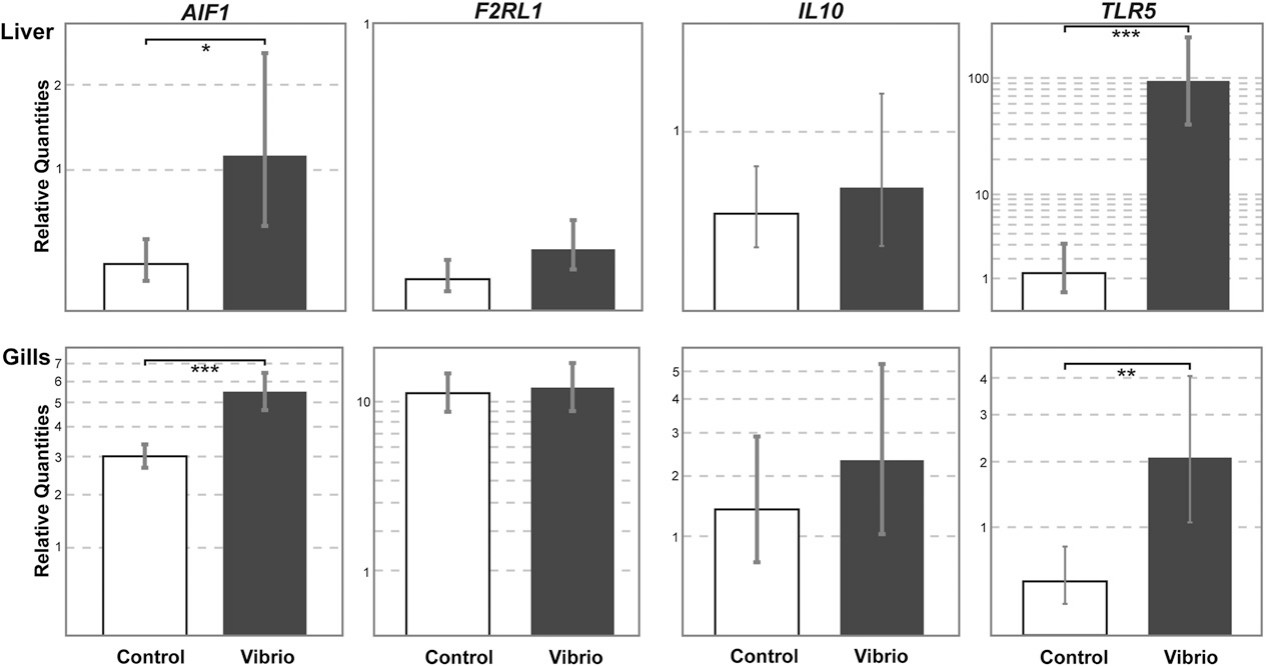
Gene expression assays for the control immune genes in *A. burtoni* adults. The relative gene expression levels (relative quantities) of *AIF1*, *F2RL1*, *IL10*, and *TLR5* measured in liver (top) and gills (bottom) for the control treatment (white bars) and experimental treatment (*Vibrio*, blue bars). *P < 0.05; **P < 0.01; ***P < 0.001. Depicted are the mean and the 95% CI (error bars).

We measured the expression levels of four control immune loci, *AIF1*, *F2RL1*, *IL10*, and *TLR5* in liver and gill tissues. These relative expression levels are depicted in Figure 4. For *AIF1* and *TLR5*, we found significantly higher levels of relative expression in liver (*P* = 0.014 and *P* < 0.001; unpaired *t* test) and gills (*P* < 0.001 and *P* = 0.006) in the experimental treatment group.

**Figure 5:**
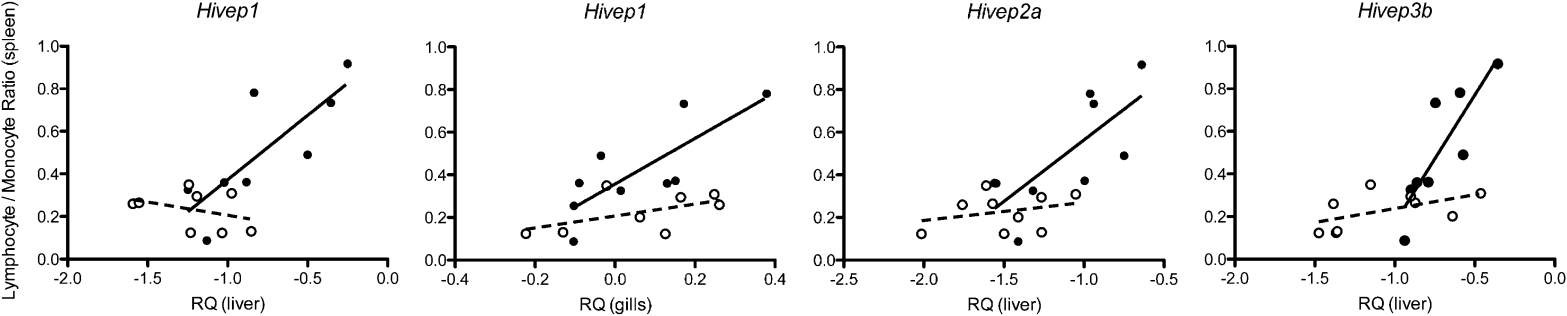
Correlations between immune response measurements and *Hivep* gene expression levels in *A. burtoni* adults. The relative gene expression levels [relative quantities (RQ)] of *Hivep1*, *Hivep2a*, and *Hivep3b* measured in gills and/or liver correlated with the observed lymphocyte/monocyte ratios measured in spleen for the control treatment (open circles and dashed fitted trend) and experimental treatment (closed circles and black fitted trend lines). Significant correlations were only observed for the experimental group: *Hivep1* (liver; Pearson *r* = 0.798, *P* = 0.018); *Hivep1* (gills; Pearson *r* = 0.794, *P* = 0.011); *Hivep2a* (Pearson *r* = 0.745, *P* = 0.021); and *Hivep3b* (Pearson *r* = 0.852, *P* = 0.007).

To analyze the effect of *Vibrio* exposure on the expression levels of the *Hivep* paralogs in detail, we assessed their expression levels in relation to an immune response parameter (*i.e.*, lymphocyte/monocyte ratio) per treatment group (*i.e.*, control and experimental). Four correlations were significant between the lymphocyte/monocyte ratio of the spleen and the relative expression of *Hivep1* (liver; Pearson *r* = 0.798, *P* = 0.018), *Hivep1* (gills; Pearson *r* = 0.794, *P* = 0.011), *Hivep2a* (Pearson *r* = 0.745, *P* = 0.021), and *Hivep3b* (Pearson *r* = 0.852, *P* = 0.007) (Figure 5). In these cases, the expression level of the *Hivep* paralogs thus correlates positively with the level of the immune response parameter.

## Discussion

In this study, we examined the molecular evolutionary history of the *Hivep* gene family members in relation to their presumed immune-related function in a renowned model system for evolutionary biology, the East African cichlid fishes. We performed comparative phylogenetic analyses and detailed screens of *d*_N_/*d*_S_ ratios, analyzed putative *cis*-regulatory regions within the loci, and, in particular, investigated the expression levels of the *Hivep* paralogs after an experimental vaccination with *V. anguillarum*. We show, for the first time to our knowledge, that the *Hivep* paralogs play a putative role in the response to vaccination in fish, and that they are characterized by signatures of long-term positive selection. Our findings regarding the *Hivep* paralogs indicate that they are important candidate genes for immune-related functions in teleost fish and suggest broader implications in relation to speciation events, such as the adaptive radiations in East African cichlid fishes.

### Exposure to *V*. *anguillarum* causes an immune response in *A. burtoni*

To test whether the exposure to a vaccination with heat-killed *V. anguillarum* resulted in an upregulation of the cellular fish immune response, we measured lymphocyte/monocyte ratios, the proportions of proliferating lymphocytes, and the expression levels of four control immune genes with demonstrated functions in the inflammatory response and immunity (Watano *et al.* 2001; Janeway and Medzhitov 2002; Akira *et al.* 2006; Jankovic and Trinchieri 2007; Rothmeier and Ruf 2012) (see *Materials and Methods* section). Consistent with an elevated immune response upon *Vibrio* vaccination, we found an increased lymphocyte production in the head kidney, the organ where clonal lymphocyte production takes place (Rombout *et al.* 2005; Abdel-Aziz *et al.* 2010). We also found a higher proportion of lymphocytes both in blood and spleen, indicating lymphocyte migration toward peripheral organs. Although lymphocytes are transported via blood, the spleen is the major lymphoid tissue associated with the clearance of blood-borne antigens (Press and Evensen 1999; Whyte 2007). Finally, the significant upregulation of both *AIF1* and *TLR5* in the *Vibrio*-exposed group indicates activation of the immune system (Watano *et al.* 2001; Janeway and Medzhitov 2002; Akira *et al.* 2006). However, we did not find a significant upregulation for two other immune genes with demonstrated functions in the immune response, *F2RL1* and *IL10* (Déry *et al.* 1998; Savan *et al.* 2003; Rothmeier and Ruf 2012). These were possibly missed by the choice of our measuring time point (21 hr after vaccination), as suggested by Savan *et al.* (2003), who found elevated *IL10* expression in carp liver tissue after LPS stimulation only within the first 6 hr of incubation.

### *Hivep* expression levels correlate with cellular immune response parameters in an East African cichlid fish

Although several functions of the *Hivep* paralogs have been demonstrated in the fruitfly, *Xenopus* frog, human, and mouse (Wu 2002), the *Hivep* paralogs have, so far, not been examined in teleost fishes. We tested, for the first time, whether there is a correlation between immune response parameters and the expression level of the *Hivep* paralogs in fish as an indicator of putative function(s) in the immune response.

Although our study does not determine the exact function of the *Hivep* paralogs within the immune response, the positive correlations between the lymphocyte/monocyte ratio and the expression levels of three *Hivep* paralogs indicate that the expression of —at least—*Hivep1*, *Hivep2a*, and *Hivep3b* is upregulated upon the experimental vaccination. This implies that *Hivep* paralogs play a role during the immune response of fish. These results provide, to our knowledge, the first indication of an immunological function of the *Hivep* paralogs in teleost fish, which is congruent with preliminary findings in pipefish (O. Roth, personal communication). The *Hivep* gene family thus offers a potential novel family of immune genes for teleost fish that, when their functions are characterized in more detail, can be used in future immunological screens.

### Other functional implications

The experimental vaccination did not lead to upregulated expression levels of all five *Hivep* paralogs. We found no correlation between the expression levels of *Hivep2b* and *Hivep3a* and the immune response measurements. These paralogs either have immunological functions beyond the scope of our experimental design or are not involved in the immune response in teleost fishes. Previously, it had been shown that *Hivep* genes are involved in functions other than the immune system in insects and vertebrates, *e.g.*, in murine osteoclastogenesis (Liu *et al.* 2011), in *BMP*/*Dpp* signaling (Grieder *et al.* 1995; Dai *et al.* 2000; Marty *et al.* 2000; Torres-Vazquez *et al.* 2001; Yao 2006; Saita *et al.* 2007; Yin *et al.* 2010), and in the development of the nervous system (Campbell and Levitt 2003; Takagi *et al.* 2006). Interestingly, several of the potential transcription factor binding sites identified within the observed CNEs correspond with these known functions of *Hivep* paralogs. For instance, we found multiple hits for components of the *BMP* signaling pathway, as well as several other developmental patterning loci, suggesting a putative role of the *Hivep* paralogs in developmental patterning and bone formation in cichlid fishes. *Hivep* paralogs have been found to play a role in the specification of *Drosophila* wing and halter discs (Torres-Vazquez *et al.* 2001), multiple *dpp*-dependent patterning events of both *Drosophila* ectoderm and mesoderm (Arora *et al.* 1995), and male tail patterning in *Caenorhabditis elegans* (Liang *et al.* 2007). The roles of *Hivep* in the *BMP* pathway, possibly through alternative splicing (Hicar *et al.* 2001; Hong and Wu 2005; Yin *et al.* 2010), together with several indications of functions in appendage specification and patterning make them candidate genes for fin patterning and anal fin egg-spot formation, a sexually selected trait involved in courtship and spawning behavior and intrasexual communication of haplochromine cichlid species (Wickler 1962; Hert 1989; Salzburger *et al.* 2005; Salzburger 2009; Theis *et al.* 2012). Future detailed expression and functional analyses should elucidate whether the *Hivep* paralogs are involved the development of egg spots in haplochromine cichlid fishes.

### Implications of positive selection on *Hivep* paralogs, immune genes, and speciation events

Positive selection, or adaptive sequence evolution, is the hallmark of evolutionary change and molecular adaptation. By comparing the nonsynonymous substitution rate (*d*_N_) to the synonymous substitution rate (*d*_S_) of protein coding genes, the selection regime (*i.e.*, neutral, purifying or positive) per amino acid can be inferred (Yang and Bielawski 2000). This method is widely used and has led to the identification of many cases of positive selection (Yang and Bielawski 2000). Genes involved in (evading) defensive systems or immunity are typically found with signatures of positive selection (Endo *et al.* 1996; Yang and Bielawski 2000; Schlenke and Begun 2003; Nielsen 2005; Nielsen *et al.* 2005; Biswas and Akey 2006; Yang 2006; Jiggins and Kim 2007; Montoya-Burgos 2011). As discussed, several functions within the immune response have been described for the *Hivep* paralogs in other species, and our detailed inferences of the *d*_N_/*d*_S_ ratios provide evidence for positive selection acting on four out of five *Hivep* paralogs. Interestingly, signs of positive selection have been found before in vertebrate *Hivep* paralogs. *Hivep2* has been found with a signature of positive selection in *Tetraodon* (Montoya-Burgos 2011) and the cow lineage (Toll-Riera *et al.* 2011), whereas *Hivep3* showed signs of positive selection in the human lineage (Vamathevan *et al.* 2008). At least for the human *Hivep* paralog, it has been suggested that the immune function is the cause for the signature of positive selection. Together, these results indicate that it is likely that the immune-related functions of the *Hivep* paralogs are the cause for the elevated *d*_N_/*d*_S_ ratios observed across vertebrate lineages, including the 14 cichlid lineages examined here.

Positively selected genes are typically only loosely connected to reproductive isolation in *Drosophila* (Wu and Ting 2004). This is in contrast to the vertebrate MHC loci, known for their signatures of positive selection (Hughes and Nei 1988; Hughes and Nei 1989; Yang and Bielawski 2000; Montoya-Burgos 2011), which have been proposed as pleiotropic speciation genes (Eizaguirre *et al.* 2009). Because these genes are involved in adaptation to novel habitats in response to different pathogenic communities and assortative mating via mate choice, their pleiotropic effects are hypothesized to induce and accelerate speciation (Eizaguirre *et al.* 2009). Our work shows that several of the *Hivep* paralogs also have putative pleiotropic roles in immune defense and an important sexually selected trait—the anal fin egg-spot in East African haplochromine cichlids—subject to mate choice (Hert 1989; Couldridge 2002; but see Theis *et al.* 2012). Mate choice for the most attractive male anal fin could thus select a certain *Hivep* genotype and thereby facilitate adaptation to pathogenic environments at the same time. Similar to the MHC loci, the *Hivep* paralogs might have played important roles during the explosive speciation events of cichlid fishes and therefore are exciting new putative speciation genes.

### *Hivep3b*: Selective patterns in haplochromines and other derived cichlid lineages

That we found evidence of lineage-specific positive selection acting on *Hivep3b* indicates that this locus underwent adaptive protein evolution in both the derived cichlid lineages, including the lamprologines, eretmodines, and the C-lineage, and the most species-rich cichlid lineage, the haplochromines. Adaptive protein evolution underlies the adaptive evolution of traits and is thus ultimately responsible for species divergence and evolutionary innovation (Yang 2006). Interestingly, the elevated *d*_N_/*d*_S_ ratios were observed in lineages that are characterized by explosive speciation and diversification events (Salzburger *et al.* 2005; Day *et al.* 2008), which can be seen as further support for the hypothesis that the pleiotropic functions of the *Hivep* paralogs —*Hivep3b* specifically— can be linked to speciation events. During such events genes could have been recruited to perform altered functions to generate novel or modified traits, which ultimately may have played a role in the divergence between species. A lineage-specific amino acid substitution in *Hivep3b* was observed for all the haplochromines (position 112 G → R) and the derived lineages (position 87 E → D), as well as several substitutions in a subset of the species belonging to these lineages. Functional analyses are now needed to test whether these substitutions have a fitness advantage for these species and, above all, their function in these cichlid lineages.

## Supplementary Material

Supporting Information
