# Supplementary material for: Immune-Related Functions of the Hivep Gene Family in East African Cichlid Fishes
Source: G3 (Bethesda). 2013 Oct 18;3(12):2205–17. doi: 10.1534/g3.113.008839 (PMC3852383; doi:10.1534/g3.113.008839)
Supplement: Supporting Information [file supp_3_12_2205__index.html]

Immune-Related Functions of the Hivep Gene Family in East African Cichlid Fishes — Supporting Information 

# Immune-Related Functions of the *Hivep* Gene Family in East African Cichlid Fishes

## Supporting Information for Diepeveen, Roth, and Salzburger, 2013

**Files in this Data Supplement:**

- Supporting Information - Figures S1-S2 and Tables S1-S3 (PDF, 1 MB)
- Figure S1 - Vistaplots for four *Hivep* paralogs. (PDF, 464 KB)
- Figure S2 - Overview of the radical amino acid properties identified by the TreeSAAP analyses for four *Hivep* paralogs. (PDF, 451 KB)
- Table S1 - Species names, Tribes and GenBank accession numbers of the five sequenced *Hivep* paralogs. (PDF, 451 KB)
- Table S2 - Ensemble accession numbers or genomic location of teleost *Hivep* sequences used for primer design, phylogenetic and/or Vista analyses. (PDF, 432 KB)
- Table S3 - Primer names and Sequences used for Sequencing (top) and Gene-expression assay (bottom). (PDF, 363 KB)
