# Supplementary material for: Immune-Related Functions of the Hivep Gene Family in East African Cichlid Fishes
Source: G3 (Bethesda). 2013 Oct 18;3(12):2205–17. doi: 10.1534/g3.113.008839 (PMC3852383; doi:10.1534/g3.113.008839)
Supplement: Supporting Information [file supp_g3.113.008839_008839SI.pdf]

G3 · Genes | Genomes | Genetics

Immune-related functions of the Hivep gene family in East African cichlid fishes

EVELINE T. DIEPEVEEN*[1], OLIVIA ROTH§ and WALTER SALZBURGER*

**Zoological Institute, University of Basel, 4051 Basel, Switzerland*
§Evolutionary Ecology of Marine Fishes, Helmholtz Centre of Ocean Research Kiel (GEOMAR), D-24105 Kiel, Germany
[1] Present address: Department of Environmental Science, Policy and Management, University of California, Berkeley, CA 94720

All generated cichlid *Hivep* sequences have been deposited into GenBank [GenBank: KF049218 - KF049416]

DOI: 10.1534/g3.113.008839

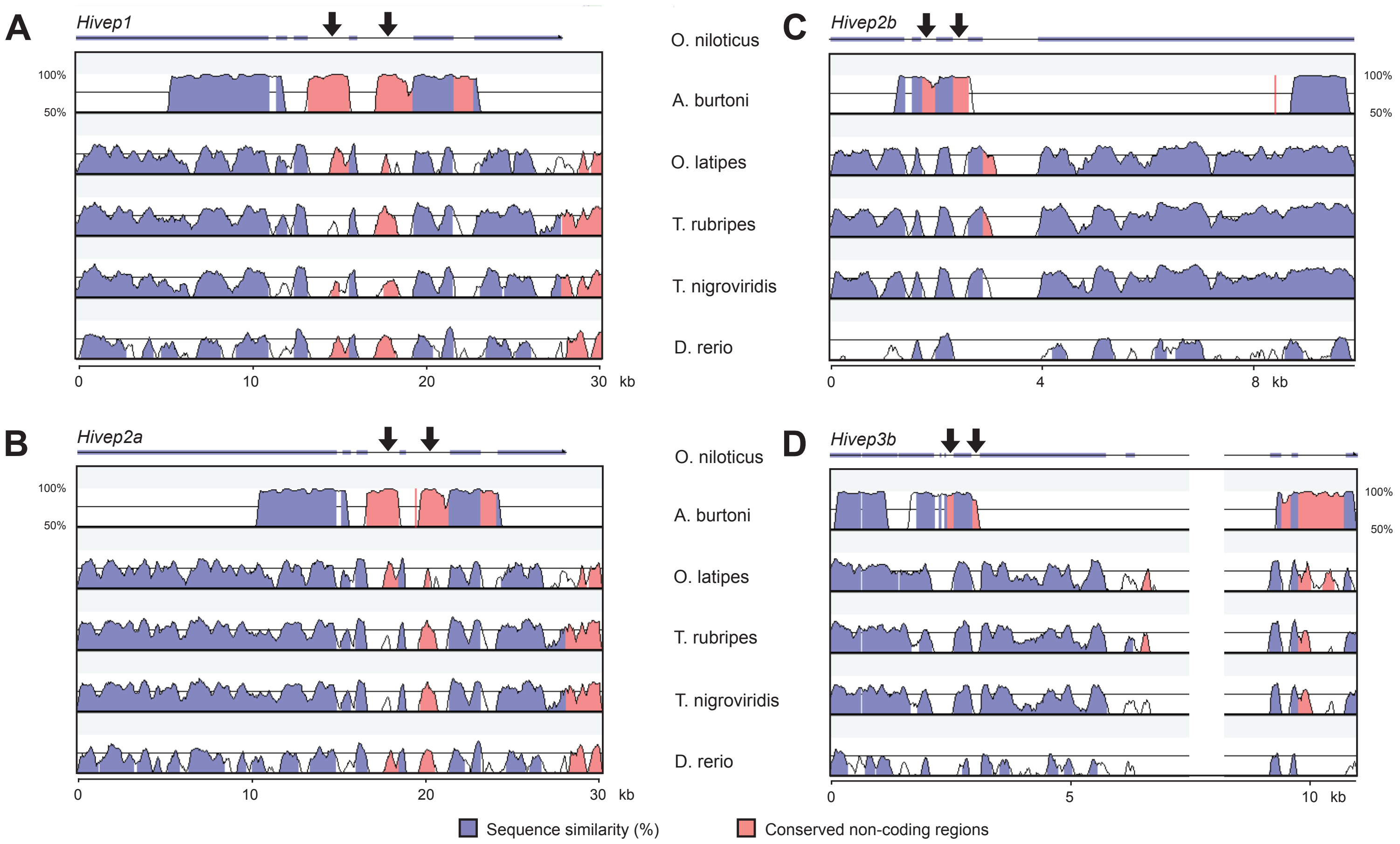

Figure S1 Vistaplots for four *Hivep* paralogs. Sequence similarity with a minimum of 50% identity between *A. burtoni* (top panel), *O. latipes* (second panel), *T. rubripes* (mid panel), *T. nigroviridis* (fourth panel), *D. rerio* (bottom panel) and *O. niloticus* (exon-intron structure on top of figure) are displayed in purple. Pink regions indicate conserved non-coding regions (CNEs). Arrows indicate the two identified CNEs in all four paralogs. Three CNEs were observed in *Hivep1* (A) and *Hivep2a* (B) in *A.burtoni*, of which two were commonly observed among teleosts. (C) Two cichlid-specific CNEs were observed in *Hivep2b*. (D) In *Hivep3b* four CNEs were observed.

Color key for radical amino acid properties per *Hivep* paralog in Figure 2

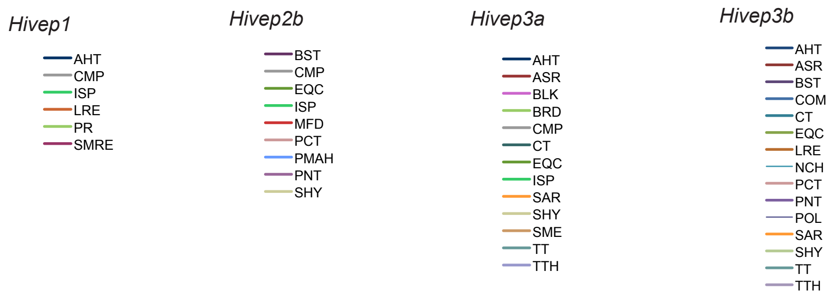

AHT=Alpha-helical tendencies
ASR=Average number of surrounding residues
BLK=Bulkiness
BRD=Buriedness
BST=Beta-structure tendencies
CMP=Compressibility
COM=Composition
CT=Coil tendencies
EQC=Equilibrium constant (ionization of COOH)
ISP=Isoelectric point
LRE=Long-range non-bonded energy
MFD=Mean r.m.s. fluctuation displacement
NCH=Normalized consensus hydrophobicity
PCT=Power to be at the C-terminal
PMAH=Power to be at the middle of alpha-helix
PNT=Power to be at the N-terminal
POL=Polarity
PR=Polar requirement
SAR=Solvent accessible reduction ratio
SHY=Surrounding hydrophobicity
SMRE=Short and medium range non-bonded energy
TT=Turn tendencies
TTH=Thermodynamic transfer hydrophobicity

Figure S2 Overview of the radical amino acid properties identified by the TreeSAAP analyses for four *Hivep* paralogs. Colors correspond to the sliding window plots in Fig. 2 and amino acid properties abbreviations are displayed for each paralog individually.

Table S1 Species names, Tribes and GenBank accession numbers of the five sequenced *Hivep* paralogs

Species	Tribe	*Hivep1*	*Hivep2a*	*Hivep2b*	*Hivep3a*	*Hivep3b*
Bathybates graueri	Bathybatini	KF049218	KF049276	KF049316	KF049357	KF049398
Benthochromis tricoti	Benthochromis	KF049245	KF049264	KF049303	KF049373	KF049385
Boulengerochromis microlepis	Boulengerochromini	KF049229	KF049258	KF049297	KF049337	KF049377
Ctenochromis benthicola	Cyphotilapiini	KF049241	KF049267	KF049306	KF049347	KF049388
Cyphotilapia frontosa	Cyphotilapiini	KF049237	KF049293	KF049333	KF049371	KF049415
Cyprichromis leptosoma	Cyprichromini	KF049227	KF049265	KF049304	KF049345	KF049386
Aulocranus dewindtii	Ectodini	KF049244	KF049263	KF049335	KF049344	KF049384
Callochromis macrops	Ectodini	KF049228	KF049268	KF049307	KF049348	KF049389
Cyathopharynx furcifer	Ectodini	KF049238	KF049296	KF049336	KF049341	KF049381
Grammatotria lemairii	Ectodini	KF049249	KF049272	KF049311	KF049352	KF049393
Xenotilapia flavipinnis	Ectodini	KF049256	KF049288	KF049328	KF049366	KF049410
Xenotilapia spiloptera	Ectodini	KF049254	KF049286	KF049326	KF049364	KF049408
Eretmodus cyanostictus	Eretmodini	KF049239	KF049295	KF049298	KF049338	KF049378
Astatotilapia burtoni	Haplochromini	KF049240	KF049260	KF049300	KF049340	KF049380
Ctenochromis horei	Haplochromini	KF049246	KF049290	KF049330	KF049368	KF049412
Altolamprologus fasciatus	Lamprologini	KF049243	KF049289	KF049329	KF049367	KF049411
Julidochromis ornatus	Lamprologini	KF049248	KF049271	KF049310	KF049351	KF049392
Lamprologus lemairii	Lamprologini	KF049255	KF049273	KF049312	KF049353	KF049394
Lepidiolamprologus elongatus	Lamprologini	KF049242	KF049294	KF049334	KF049372	KF049416
Neolamprologus furcifer	Lamprologini	KF049230	KF049275	KF049315	KF049356	KF049397
Neolamprologus pulcher	Lamprologini	KF049231	KF049274	KF049314	KF049355	KF049396
Neolamprologus sexfasciatus	Lamprologini	KF049250	-*	KF049313	KF049354	KF049395
Neolamprologus tetracanthus	Lamprologini	KF049251	KF049278	KF049318	KF049374	KF049400
Telmatochromis dhonti	Lamprologini	KF049257	KF049284	KF049324	KF049375	KF049406
Variabilichromis moorii	Lamprologini	KF049219	KF049292	KF049332	KF049370	KF049414
Gnathochromis permaxillaris	Limnochromini	KF049232	KF049269	KF049308	KF049349	KF049390
Limnochromis staneri	Limnochromini	KF049220	KF049262	KF049302	KF049343	KF049383
Reganochromis calliurus	Limnochromini	KF049252	KF049282	KF049322	KF049376	KF049404
Haplotaxodon microlepis	Perissodini	KF049247	KF049270	KF049309	KF049350	KF049391
Perissodus microlepis	Perissodini	KF049233	KF049279	KF049319	KF049359	KF049401
Plecodus straeleni	Perissodini	KF049221	KF049261	KF049301	KF049342	KF049382
Oreochromis tanganicae	Tilapiini	KF049234	KF049277	KF049317	KF049358	KF049399
Trematocara nigrifrons	Trematocarini	KF049235	KF049280	KF049320	KF049360	KF049402
Lobochilotes labiatus	Tropheini	KF049222	KF049287	KF049327	KF049365	KF049409
Petrochromis famula	Tropheini	KF049236	KF049281	KF049321	KF049361	KF049403
Petrochromis polyodon	Tropheini	KF049253	KF049283	KF049323	KF049362	KF049405
Pseudosimochromis curvifrons	Tropheini	KF049223	KF049259	KF049299	KF049339	KF049379
Simochromis diagramma	Tropheini	KF049224	KF049291	KF049331	KF049369	KF049413
Tropheus moori	Tropheini	KF049225	KF049266	KF049305	KF049346	KF049387
Tylochromis polylepis	Tylochromini	KF049226	KF049285	KF049325	KF049363	KF049407

* The *Hivep2a* sequence for *N. sexfasciatus* could not be submitted to GenBank, due to a lack of completeness

Table S2 Ensemble accession numbers or genomic location of teleost *Hivep* sequences used for primer design, phylogenetic and/or Vista analyses

**Species**	**Ensemble/GenBank accession numbers and preliminary genome information**				
	Hivep1	***Hivep2a***	***Hivep2b***	***Hivep3a***	***Hivep3b***
Danio rerio	ENSDARG00000079528	ENSDARG00000039987	ENSDARG00000018773	ENSDARG00000075928	ENSDARG00000037154
Gadus morhua				ENSGMOG00000005293	
Gasterosteus aculeatus		ENSGACG00000011974	ENSGACG00000011743		ENSGACG00000007350
Tetraodon nigrovirdis	ENSTNIG00000004491	ENSTNIG00000010718	ENSTNIG00000019322		ENSTNIG00000009667
Takifugu rubripes	scaffold_69:866682-876734:1	ENSTRUG00000015889	ENSTRUG00000007039	ENSTRUG00000010896	ENSTRUG00000013983
Oryzias latipes	ENSORLG00000009270	ENSORLG00000018133	ENSORLG00000008843		ENSORLG00000019868
Oreochromis niloticus	ENSONIG00000013692	ENSONIG00000018691	ENSONIG00000000567		ENSONIG00000006743
Astatotilapia burtoni[1]			scaffold_477	scaffold_91	
Neolamprologus brichardi[1]			scaffold_57	scaffold_36	
Pundamilia nyererei[1]			scaffold_69	scaffold_147	

[1] v1 assembly of the preliminary whole genome sequences by the Cichlid Genome Consortium (cichlid.umd.edu/CGCindex.html)

Table S3 Primer names and Sequences used for Sequencing (top) and Gene-expression assay (bottom)

Locus	Forward primer	5' - 3' Sequence	Reverse Primer	5' - 3' Sequence
Hivep1	Hivep1_2F93	CGAGAGAACATCCAAAACAAAC	Hivep1_C4R74	ATCCCACATTCCTCACAGA
	Hivep1_4F105	AACACATCCGCACCCACTC	Hivep1_5R31	GCCTTGGATTTCATGTGCTT
	Hivep1_4F144	GCGTCCACTGCAACTTCTC	Hivep1_5R59	CCCCATCTCCAAGCATTTC
	Hivep1_5F7	TGACCAAGCACATGAAATCC	Hivep1_6R471	GGCACATCAAAGTCAGGGTAG
	Hivep1_6F159	CAGCACATCTCTTCCAAA	Hivep1_7R131	GGTCTCTGTTCCTGGGGTTT
Hivep2a	Hivep2a_1F73	CCCAGCTCNTTTGGACAGTA	Hivep2a_2R484	AGGTTAGGAGGGGTGGAAGT
	Hivep2a_2F219	GCCTGACTACCCTGAAGCAA	Hivep2a_3R133	TTTCAGGCAACTCCATAGGC
	Hivep2a_8F129	CCGACCCTATGTCTGCAAGT	Hivep2a_9R95	GTCTGCCTCAGTGTCCTCAA
Hivep2b	Hivep2b_1F6	AGCAGAAACCACAAAAACCT	Hivep2b_1R500	CTTGATAGGACTTTGCGTTTG
	Hivep2b_2F42	CGGGAAGAAGGTGAAAGACA	Hivep2b_5R129	CGTCCTCATCGTCATCTTCA
Hivep3a	Hivep3a_1F1	CAGAAACGTGAGCGTAAG	Hivep3a_1R936	ATTTGARGGTGGGTGATGAA
	Hivep3a_1F856	GTTGGCGATGTGGGCTAT	Hivep3a_1R841	TGATGTTTTGCTGTGGTGGT
	Hivep3a_1F1297	TGCCTAKCACATCAACCCATC	Hivep3a_1R2243	CTTTCYCTCCACCCAAYTGC
Hivep3b	Hivep3b_1F8	GAGAAGAAACCACAAAAAC	Hivep3b_1R123	GGCTTCTCCTTTGGGATCA
	Hivep3b_1F132	AGCCTGCCATAGAGGTTCC	Hivep3b_4R50	AATGTCTGACTTCGCTGAGG
	Hivep3b_F40	CGGCAGGGGAAAGTACATA	Hivep3b_R223	CCTTGCTTAGAGTGCCTGCT

Locus	Forward sequence	Reverse sequence
Hivep1	ATGCCCGCAGCAGGAA	TCCATGTAGATGTCCAAGCTGTTT
Hivep2a	TGCCATGTCCCAGTCACTTAA	GAGCGTGAAAAGTAGCCAGAGTCT
Hivep2b	CGAGGCTAGTCGTGCAATTCA	AGAGTCACTGCTTCTTTTTTCAGACA
Hivep3a	GAGCAGCCAGCTAGCCAAAC	ATAAACTGGCTTCAGGTGGCATT
Hivep3b	CCAGACAGCCACAGCAACAA	CCCCACATGTTCCACATTCA
EF1	GCCCCTGCAGGACGTCTA*	CGGCCGACGGGTACAGT*
RpSA3	AGACCAATGACCTGAAGGAAGTG*	TCTCGATGTCCTTGCCAACA*
AIF1	GGTTTGAAGAGGATGTTGGAGAA	CAACCACCTCTGCCATCATTT
IL10	TGGCGCTCGCGTCTTT	CGGCAGCAGCGGTTGT
F2RL1	TGGGAATAAAGCACACAAGGAA	CATCAAAGAACCGACACAAAGTTG
TLR5	GCATGTTGCTGGTCTCAATTTG	GAAGTGAGGCCCTTGAAAACAC

* Primers from Colombo *et al*. 2013
