# Supplementary material for: Immune-Related Functions of the Hivep Gene Family in East African Cichlid Fishes
Source: G3 (Bethesda). 2013 Oct 18;3(12):2205–17. doi: 10.1534/g3.113.008839 (PMC3852383; doi:10.1534/g3.113.008839)
Supplement: Supporting Information [file supp_g3.113.008839_FigureS1.pdf]

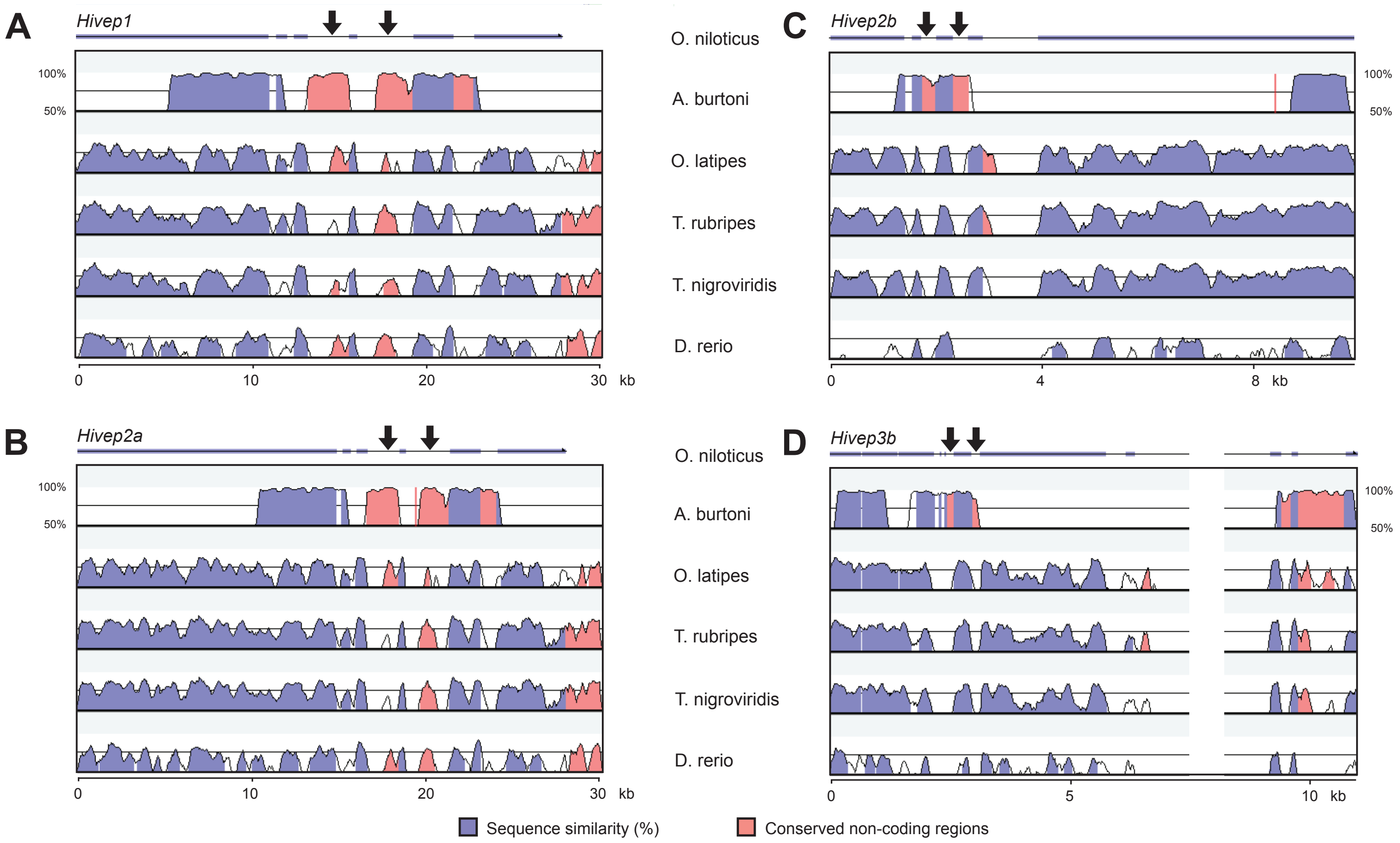

Figure S1 Vistaplots for four *Hivep* paralogs. Sequence similarity with a minimum of 50% identity between *A. burtoni* (top panel), *O. latipes* (second panel), *T. rubripes* (mid panel), *T. nigroviridis* (fourth panel), *D. rerio* (bottom panel) and *O. niloticus* (exon-intron structure on top of figure) are displayed in purple. Pink regions indicate conserved non-coding regions (CNEs). Arrows indicate the two identified CNEs in all four paralogs. Three CNEs were observed in *Hivep1* (A) and *Hivep2a* (B) in *A.burtoni*, of which two were commonly observed among teleosts. (C) Two cichlid-specific CNEs were observed in *Hivep2b*. (D) In *Hivep3b* four CNEs were observed.
