# Supplementary material for: Immune-Related Functions of the Hivep Gene Family in East African Cichlid Fishes
Source: G3 (Bethesda). 2013 Oct 18;3(12):2205–17. doi: 10.1534/g3.113.008839 (PMC3852383; doi:10.1534/g3.113.008839)
Supplement: Supporting Information [file supp_g3.113.008839_FigureS2.pdf]

Color key for radical amino acid properties per *Hivep* paralog in Figure 2

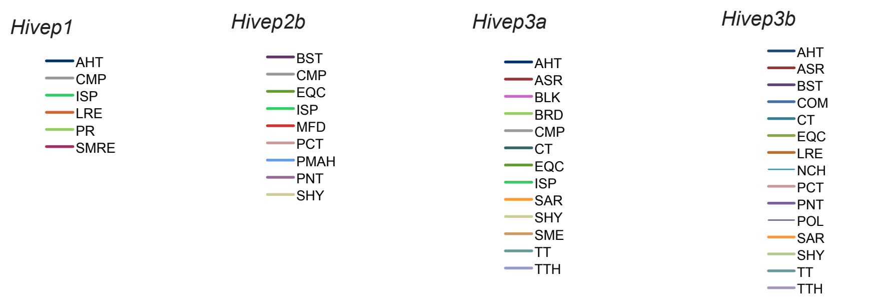

AHT=Alpha-helical tendencies
ASR=Average number of surrounding residues
BLK=Bulkiness
BRD=Buriedness
BST=Beta-structure tendencies
CMP=Compressibility
COM=Composition
CT=Coil tendencies
EQC=Equilibrium constant (ionization of COOH)
ISP=Isoelectric point
LRE=Long-range non-bonded energy
MFD=Mean r.m.s. fluctuation displacement
NCH=Normalized consensus hydrophobicity
PCT=Power to be at the C-terminal
PMAH=Power to be at the middle of alpha-helix
PNT=Power to be at the N-terminal
POL=Polarity
PR=Polar requirement
SAR=Solvent accessible reduction ratio
SHY=Surrounding hydrophobicity
SMRE=Short and medium range non-bonded energy
TT=Turn tendencies
TTH=Thermodynamic transfer hydrophobicity

Figure S2 Overview of the radical amino acid properties identified by the TreeSAAP analyses for four *Hivep* paralogs. Colors correspond to the sliding window plots in Fig. 2 and amino acid properties abbreviations are displayed for each paralog individually.
