# Supplementary material for: Immune-Related Functions of the Hivep Gene Family in East African Cichlid Fishes
Source: G3 (Bethesda). 2013 Oct 18;3(12):2205–17. doi: 10.1534/g3.113.008839 (PMC3852383; doi:10.1534/g3.113.008839)
Supplement: Supporting Information [file supp_g3.113.008839_TableS1.pdf]

Table S1 Species names, Tribes and GenBank accession numbers of the five sequenced *Hivep* paralogs

Species	Tribe	*Hivep1*	*Hivep2a*	*Hivep2b*	*Hivep3a*	*Hivep3b*
Bathybates graueri	Bathybatini	KF049218	KF049276	KF049316	KF049357	KF049398
Benthochromis tricoti	Benthochromis	KF049245	KF049264	KF049303	KF049373	KF049385
Boulengerochromis microlepis	Boulengerochromini	KF049229	KF049258	KF049297	KF049337	KF049377
Ctenochromis benthicola	Cyphotilapiini	KF049241	KF049267	KF049306	KF049347	KF049388
Cyphotilapia frontosa	Cyphotilapiini	KF049237	KF049293	KF049333	KF049371	KF049415
Cyprichromis leptosoma	Cyprichromini	KF049227	KF049265	KF049304	KF049345	KF049386
Aulocranus dewindtii	Ectodini	KF049244	KF049263	KF049335	KF049344	KF049384
Callochromis macrops	Ectodini	KF049228	KF049268	KF049307	KF049348	KF049389
Cyathopharynx furcifer	Ectodini	KF049238	KF049296	KF049336	KF049341	KF049381
Grammatotria lemairii	Ectodini	KF049249	KF049272	KF049311	KF049352	KF049393
Xenotilapia flavipinnis	Ectodini	KF049256	KF049288	KF049328	KF049366	KF049410
Xenotilapia spiloptera	Ectodini	KF049254	KF049286	KF049326	KF049364	KF049408
Eretmodus cyanostictus	Eretmodini	KF049239	KF049295	KF049298	KF049338	KF049378
Astatotilapia burtoni	Haplochromini	KF049240	KF049260	KF049300	KF049340	KF049380
Ctenochromis horei	Haplochromini	KF049246	KF049290	KF049330	KF049368	KF049412
Altolamprologus fasciatus	Lamprologini	KF049243	KF049289	KF049329	KF049367	KF049411
Julidochromis ornatus	Lamprologini	KF049248	KF049271	KF049310	KF049351	KF049392
Lamprologus lemairii	Lamprologini	KF049255	KF049273	KF049312	KF049353	KF049394
Lepidiolamprologus elongatus	Lamprologini	KF049242	KF049294	KF049334	KF049372	KF049416
Neolamprologus furcifer	Lamprologini	KF049230	KF049275	KF049315	KF049356	KF049397
Neolamprologus pulcher	Lamprologini	KF049231	KF049274	KF049314	KF049355	KF049396
Neolamprologus sexfasciatus	Lamprologini	KF049250	-*	KF049313	KF049354	KF049395
Neolamprologus tetracanthus	Lamprologini	KF049251	KF049278	KF049318	KF049374	KF049400
Telmatochromis dhonti	Lamprologini	KF049257	KF049284	KF049324	KF049375	KF049406
Variabilichromis moorii	Lamprologini	KF049219	KF049292	KF049332	KF049370	KF049414
Gnathochromis permaxillaris	Limnochromini	KF049232	KF049269	KF049308	KF049349	KF049390
Limnochromis staneri	Limnochromini	KF049220	KF049262	KF049302	KF049343	KF049383
Reganochromis calliurus	Limnochromini	KF049252	KF049282	KF049322	KF049376	KF049404
Haplotaxodon microlepis	Perissodini	KF049247	KF049270	KF049309	KF049350	KF049391
Perissodus microlepis	Perissodini	KF049233	KF049279	KF049319	KF049359	KF049401
Plecodus straeleni	Perissodini	KF049221	KF049261	KF049301	KF049342	KF049382
Oreochromis tanganicae	Tilapiini	KF049234	KF049277	KF049317	KF049358	KF049399
Trematocara nigrifrons	Trematocarini	KF049235	KF049280	KF049320	KF049360	KF049402
Lobochilotes labiatus	Tropheini	KF049222	KF049287	KF049327	KF049365	KF049409
Petrochromis famula	Tropheini	KF049236	KF049281	KF049321	KF049361	KF049403
Petrochromis polyodon	Tropheini	KF049253	KF049283	KF049323	KF049362	KF049405
Pseudosimochromis curvifrons	Tropheini	KF049223	KF049259	KF049299	KF049339	KF049379
Simochromis diagramma	Tropheini	KF049224	KF049291	KF049331	KF049369	KF049413
Tropheus moori	Tropheini	KF049225	KF049266	KF049305	KF049346	KF049387
Tylochromis polylepis	Tylochromini	KF049226	KF049285	KF049325	KF049363	KF049407

* The *Hivep2a* sequence for *N. sexfasciatus* could not be submitted to GenBank, due to a lack of completeness
