# Supplementary material for: Immune-Related Functions of the Hivep Gene Family in East African Cichlid Fishes
Source: G3 (Bethesda). 2013 Oct 18;3(12):2205–17. doi: 10.1534/g3.113.008839 (PMC3852383; doi:10.1534/g3.113.008839)
Supplement: Supporting Information [file supp_g3.113.008839_TableS2.pdf]

Table S2 Ensemble accession numbers or genomic location of teleost *Hivep* sequences used for primer design, phylogenetic and/or Vista analyses

Species	Ensemble/GenBank accession numbers and preliminary genome information				
	Hivep1	***Hivep2a***	***Hivep2b***	***Hivep3a***	***Hivep3b***
Danio rerio	ENSDARG00000079528	ENSDARG00000039987	ENSDARG00000018773	ENSDARG00000075928	ENSDARG00000037154
Gadus morhua				ENSGMOG00000005293	
Gasterosteus aculeatus		ENSGACG00000011974	ENSGACG00000011743		ENSGACG00000007350
Tetraodon nigrovirdis	ENSTNIG00000004491	ENSTNIG00000010718	ENSTNIG00000019322		ENSTNIG00000009667
Takifugu rubripes	scaffold_69:866682-876734:1	ENSTRUG00000015889	ENSTRUG00000007039	ENSTRUG00000010896	ENSTRUG00000013983
Oryzias latipes	ENSORLG00000009270	ENSORLG00000018133	ENSORLG00000008843		ENSORLG00000019868
Oreochromis niloticus	ENSONIG00000013692	ENSONIG00000018691	ENSONIG00000000567		ENSONIG00000006743
Astatotilapia burtoni[1]			scaffold_477	scaffold_91	
Neolamprologus brichardi[1]			scaffold_57	scaffold_36	
Pundamilia nyererei[1]			scaffold_69	scaffold_147	

[1] v1 assembly of the preliminary whole genome sequences by the Cichlid Genome Consortium (cichlid.umd.edu/CGCindex.html)
