# Supplementary material for: Immune-Related Functions of the Hivep Gene Family in East African Cichlid Fishes
Source: G3 (Bethesda). 2013 Oct 18;3(12):2205–17. doi: 10.1534/g3.113.008839 (PMC3852383; doi:10.1534/g3.113.008839)
Supplement: Supporting Information [file supp_g3.113.008839_TableS3.pdf]

Table S3 Primer names and Sequences used for Sequencing (top) and Gene-expression assay (bottom)

Locus	Forward primer	5' - 3' Sequence	Reverse Primer	5' - 3' Sequence
Hivep1	Hivep1_2F93	CGAGAGAACATCCAAAACAAAC	Hivep1_C4R74	ATCCCACATTCCTCACAGA
	Hivep1_4F105	AACACATCCGCACCCACTC	Hivep1_5R31	GCCTTGGATTTCATGTGCTT
	Hivep1_4F144	GCGTCCACTGCAACTTCTC	Hivep1_5R59	CCCCATCTCCAAGCATTTC
	Hivep1_5F7	TGACCAAGCACATGAAATCC	Hivep1_6R471	GGCACATCAAAGTCAGGGTAG
	Hivep1_6F159	CAGCACATCTCTTCCAAA	Hivep1_7R131	GGTCTCTGTTCCTGGGGTTT
Hivep2a	Hivep2a_1F73	CCCAGCTCNTTTGGACAGTA	Hivep2a_2R484	AGGTTAGGAGGGGTGGAAGT
	Hivep2a_2F219	GCCTGACTACCCTGAAGCAA	Hivep2a_3R133	TTTCAGGCAACTCCATAGGC
	Hivep2a_8F129	CCGACCCTATGTCTGCAAGT	Hivep2a_9R95	GTCTGCCTCAGTGTCCTCAA
Hivep2b	Hivep2b_1F6	AGCAGAAACCACAAAAACCT	Hivep2b_1R500	CTTGATAGGACTTTGCGTTTG
	Hivep2b_2F42	CGGGAAGAAGGTGAAAGACA	Hivep2b_5R129	CGTCCTCATCGTCATCTTCA
Hivep3a	Hivep3a_1F1	CAGAAACGTGAGCGTAAG	Hivep3a_1R936	ATTTGARGGTGGGTGATGAA
	Hivep3a_1F856	GTTGGCGATGTGGGCTAT	Hivep3a_1R841	TGATGTTTTGCTGTGGTGGT
	Hivep3a_1F1297	TGCCTAKCACATCAACCCATC	Hivep3a_1R2243	CTTTCYCTCCACCCAAYTGC
Hivep3b	Hivep3b_1F8	GAGAAGAAACCACAAAAAC	Hivep3b_1R123	GGCTTCTCCTTTGGGATCA
	Hivep3b_1F132	AGCCTGCCATAGAGGTTCC	Hivep3b_4R50	AATGTCTGACTTCGCTGAGG
	Hivep3b_F40	CGGCAGGGGAAAGTACATA	Hivep3b_R223	CCTTGCTTAGAGTGCCTGCT

Locus	Forward sequence	Reverse sequence
Hivep1	ATGCCCGCAGCAGGAA	TCCATGTAGATGTCCAAGCTGTTT
Hivep2a	TGCCATGTCCCAGTCACTTAA	GAGCGTGAAAAGTAGCCAGAGTCT
Hivep2b	CGAGGCTAGTCGTGCAATTCA	AGAGTCACTGCTTCTTTTTTCAGACA
Hivep3a	GAGCAGCCAGCTAGCCAAAC	ATAAACTGGCTTCAGGTGGCATT
Hivep3b	CCAGACAGCCACAGCAACAA	CCCCACATGTTCCACATTCA
EF1	GCCCCTGCAGGACGTCTA*	CGGCCGACGGGTACAGT*
RpSA3	AGACCAATGACCTGAAGGAAGTG*	TCTCGATGTCCTTGCCAACA*
AIF1	GGTTTGAAGAGGATGTTGGAGAA	CAACCACCTCTGCCATCATTT
IL10	TGGCGCTCGCGTCTTT	CGGCAGCAGCGGTTGT
F2RL1	TGGGAATAAAGCACACAAGGAA	CATCAAAGAACCGACACAAAGTTG
TLR5	GCATGTTGCTGGTCTCAATTTG	GAAGTGAGGCCCTTGAAAACAC

* Primers from Colombo *et al*. 2013
